# Temperature and sex ratios at birth

**DOI:** 10.1073/pnas.2422625123

**Published:** 2026-02-19

**Authors:** Jasmin Abdel Ghany, Joshua Wilde, Anna Dimitrova, Ridhi Kashyap, Raya Muttarak

**Affiliations:** ^a^Nuffield College, University of Oxford, OxfordOX1 1NF, United Kingdom; ^b^Leverhulme Centre for Demographic Science, Department of Population Health, University of Oxford, OxfordOX1 1JD, United Kingdom; ^c^Laboratory of Fertility and Well-Being, Max Planck Institute for Demographic Research, Rostock, Mecklenburg-Vorpommern18057, Germany; ^d^Population Research Center, Portland State University, Portland, OR97201; ^e^IZA Institute of Labor Economics, Bonn53113, Germany; ^f^Barcelona Institute for Global Health, Barcelona08036, Spain; ^g^Department of Sociology, University of Oxford, OxfordOX1 1JD, United Kingdom; ^h^Department of Statistical Sciences, University of Bologna, BolognaF9X2+3V, Italy; ^i^Department of Social and Political Sciences, Bocconi University, MailandC5XQ+CX, Italy

**Keywords:** sex ratios at birth, temperature, prenatal exposure, extreme heat, abortion

## Abstract

Human sex ratios at birth (SRBs) shape population composition and are closely linked to maternal health and gender discrimination. In the context of environmental change, SRBs may theoretically be skewed by physiological or behavioral responses to exposure to extreme heat. However, evidence for this is limited. In this study, we estimate the effect of prenatal exposure to temperature on birth sex by linking survey data on 5 million live births in 33 sub-Saharan African countries and India with high-resolution temperature data. To distinguish between spontaneous and induced abortions, we exploit sociodemographic differentials, exposure timing, and regional differences in son preference. We find that days with a maximum temperature above 20 °C are negatively associated with male births in both regions. In sub-Saharan Africa, we observe fewer male births after high first-trimester temperature exposure, consistent with increased spontaneous abortions from maternal heat stress. This is particularly true for births by mothers in rural areas, with little formal education, and for higher birth orders. By contrast, in India, we find that second-trimester temperature exposure is associated with fewer male births, consistent with reductions in induced sex-selective abortions. As expected, these reductions are concentrated in high birth orders and older mothers. We also find large reductions in male births by sonless mothers in northern Indian states, where son preference is greater. These findings demonstrate that heat exposure harms maternal health, increases prenatal mortality, and influences family planning behavior, leading to a complex effect on SRBs.

## Significance

While some evidence suggests that sex ratios at birth (SRBs) are shaped by environmental and social factors, little is known about the relationship between temperature and sex ratios at birth. We show that high temperatures in the nine months before birth are negatively associated with male births in sub-Saharan Africa and India. The exposure timing demonstrates that ambient heat can increase prenatal mortality in early pregnancy, particularly among males, in both world regions. We also demonstrate that in regions with high son preference, elevated temperatures during windows where sex-selective abortions could take place reduce these abortions. These findings demonstrate that heat exposure may have complex behavioral and biological implications for maternal and fetal health and ramifications on social phenomena such as gender discriminatory practices.

Human sex ratios at birth^*^ (SRBs)—or the ratio of male to female offspring—have been a fascination of social scientists from at least the 1600s (1, 2). Over the centuries, several explanations have been proposed and dismissed to explain SRB variations (1). A consensus emerged in the mid-1900s that SRBs were likely constant, genetically determined, and invariant to social or environmental shocks (1). However, since the 1970s, new theoretical biological mechanisms were proposed by which social and environmental factors could affect SRBs, most notably via male in utero fragility (3). This led to a re-emergence of research in this field (4). At the same time, interest in SRBs increased dramatically among social scientists as ultrasound technology led to a rise in sex-selective abortion of girls in many countries in Asia and Eastern Europe, skewing SRBs significantly above their natural levels (5, 6).

Yet little attention has been given to the possible effects of extreme heat on SRBs. This is particularly puzzling given the renewed interest in social, environmental, and behavioral determinants of SRBs (4, 7, 8), and the growing literature on the effect of extreme heat on unintended pregnancy terminations via heat-induced in utero mortality (9–15), which is triggered by maternal heat stress. Given existing evidence on male in utero fragility and heat-induced in utero mortality, we ask whether the prenatal mortality effects of heat are male-biased, manifesting themselves in a skew in the SRB.

**Fig. 1.**
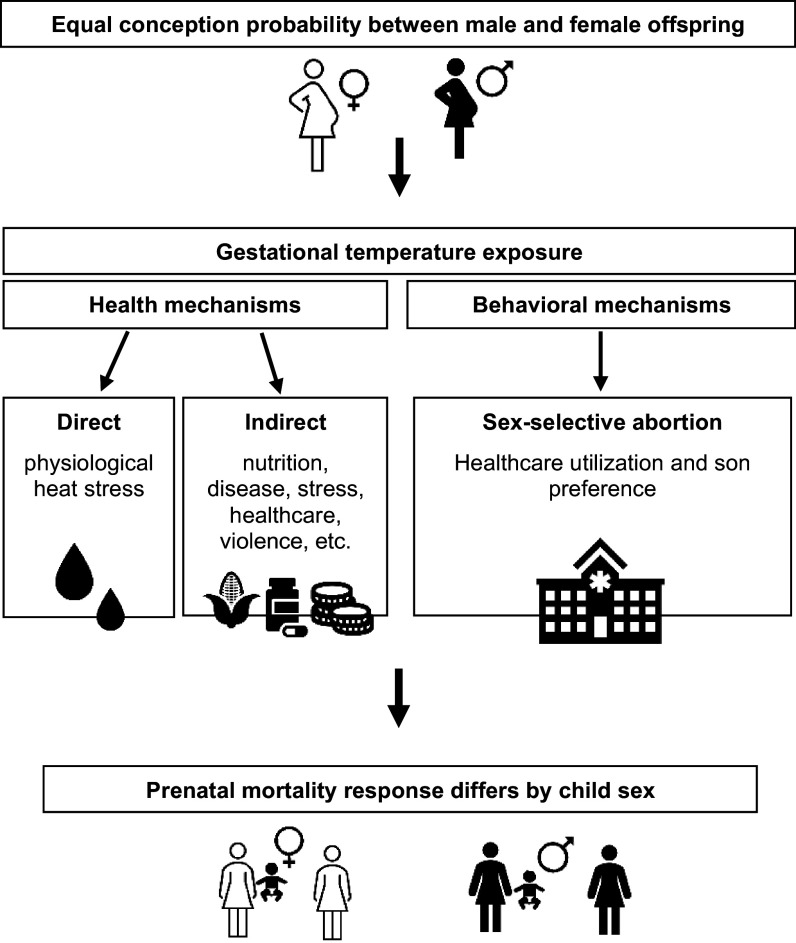
Conceptual framework on biological health and behavioral mechanisms in response to temperature exposure before birth that may cause sex-specific mortality responses.

In this paper, we estimate the effect of ambient temperature during conception and pregnancy on the sex ratio at birth.^*^ To assess both biophysical health and behavioral mechanisms (Fig. 1), we conduct a comparative analysis across two major world regions with vastly different experiences with son preference and sex-selective abortion. Specifically, we compare differences in the effect of temperature exposure between India and sub-Saharan Africa. In India, son preference is strong, and sex-selective abortion of female pregnancies has led to male-skewed SRBs (6). By contrast, in sub-Saharan Africa, there is little evidence of son preference, and sex-selective abortion is considered minimal (16–18). The present study provides a large-scale study on the impact of temperature during conception and pregnancy on human SRBs.

The sex ratio at birth (secondary sex ratio)^*^ may respond to extreme temperature via a sex-biased prenatal mortality response after conception. Orzack et al. (19) show that at conception, the sex ratio is balanced (0.5 proportion male). However, prenatal mortality introduces a sex bias that shifts throughout pregnancy. Building on this study that provides fundamental insight into early human development, we investigate how prenatal mortality changes in response to a specific prenatal stressor (temperature). Gestational heat exposure threatens the maternal body’s ability to thermoregulate, increasing the risk of pregnancy loss (20, 21). Such pregnancy losses are triggered by fetal strain that can arise from dehydration, the diversion of blood and oxygen flow away from the placenta, hormonal dysregulation, and inflammatory responses (22, 23). We hypothesize that these heat-induced pregnancy losses are male-biased, in line with Trivers and Willard’s “frail male” hypothesis (1973). According to this evolutionary argument, weak males may have a lower chance of surviving to birth under poor environmental conditions. After birth, males have lower survival probabilities than females and thus require greater maternal investment (24, 25). This makes frail male pregnancies not only costly for the mother but also less likely to yield further offspring. In consequence, to protect maternal resources and ultimately increase offspring, under environmental stress in utero selection may be more pronounced for males.

Beyond direct physiological impacts of heat on pregnancy survival, there may be reproductive behavioral responses to heat, particularly in terms of induced abortion behavior, that shape the SRB. To examine this vastly understudied behavioral mechanism, we exploit a comparison between India and sub-Saharan Africa. In most countries, an SRB of 103 to 107 boys to 100 girls is observed (6). In India, by contrast, the SRB is significantly male-skewed because of sex-selective abortions of female pregnancies, which are motivated by son preference. Particularly northern states in India have consistently shown highly inflated SRBs of around 116:100 in 2005–2016 (26–28). In southern states, which are not known for sex-selection, the SRB was estimated at around 107:100 (29). The intensity of son preference and its manifestation in prenatal sex selection are not only more dominant in northern states of India but also intensified at later birth orders and in families without sons (30–32). High temperatures could impact abortion access through mobility disruptions (33–36) or by increasing financial uncertainty and reducing income generation (37, 38). Hence, we investigate heat effects on the probability of male birth for higher-order births (four and higher) and between sonless mothers and mothers with at least one son between northern and southern states, and compare results to the sub-Saharan African placebo. We conduct this test for the second gestational trimester, as sex determination is reliable from approximately the 13th gestational week (39, 40).

Existing studies on the impact of temperature on SRBs have several methodological limitations. These studies link annual temperature measurements to births on the country level, (7, 41) which cannot a) adequately capture variation of exposures between subnational geographic areas, b) accurately map the temperature exposure at a location to the pregnancy duration of each birth on the microlevel, c) provide insight into the potential nonlinearity of the temperature–sex relationship although different temperature thresholds might have varying impacts on the SRB, and d) account for seasonality in environmental exposures, parental selection into pregnancy, underlying parental health, and other proximate and distal factors. In addition, to identify environmental determinants of the SRB, sufficient variation within and across geographic units and seasons is necessary. This has led to inconsistent findings from small-scale single-country studies (41–43) and a focus on high-income countries where large, high-quality register data are available. In the Global South, the link between temperature and SRBs remains unexplored, despite populations’ heightened vulnerability to the impacts of extreme heat.

We address these issues by pooling microlevel data on 5 million live births from 104 Demographic and Health Surveys (DHS) conducted between 2000 and 2022 in 33 countries in sub-Saharan Africa and India (44). We link each georeferenced birth with gridded, high-resolution daily maximum temperature data (45) for the approximate pregnancy period, using information on the month of birth. Our fixed-effects regressions estimate the impact of days with varying temperature intensity (below 15 °C, 20 to 25 °C, 25 to 30 °C and above 30 °C) in the three approximate pregnancy trimesters on the SRB in sub-Saharan Africa and India, while accounting for seasonality, nonlinearity, all time-constant characteristics, and time trends on the subnational regional level. To infer about specific mechanisms, we use the timing of exposure, sociodemographic differentials, and cultural differences in son preference between sub-Saharan Africa and India. Our study aims to provide insight into the relationship between temperature and SRBs and the context-specific biological and behavioral determinants underpinning it.

## Results

Our analysis draws on two separate DHS samples (44) of live births for sub-Saharan Africa^†^ (N = 2,981,905) and India (N = 1,977,013) from 104 surveys at 59,087 primary sampling units (PSUs, i.e. a village or city block).^‡^ The baseline SRB in sub-Saharan Africa is 50.87% male (103.42 males to 100 females), whereas in India, the SRB is 52.37% (109.96 males to 100 females). The PSUs in sub-Saharan Africa and India exhibit similarly high daily maximum temperatures (the mean of the daily maximum temperature in the month of birth is 30.0 °C in sub-Saharan Africa and 30.3 °C in India), but more variation between clusters in India, where the SD is 6.2 (sub-Saharan Africa 4.8). Heat days above 30 °C are the most common of all the temperature ranges—at least 33% of all gestational days fall into this range in all trimesters across both samples. The maps in *SI Appendix*, Fig. S1 show the dominant temperate, arid, and tropical climate zones in sub-Saharan Africa and India and the geographic distribution of PSUs for the samples. Most births are by mothers who live in rural areas (73.8% in sub-Saharan Africa and 79.9% in India) and mothers with no or only primary level formal education (84.3% in sub-Saharan Africa and 61.06% in India). The births are concentrated in parities three and higher in sub-Saharan Africa (56.8%) and in the first two parities in India (62.5%). Detailed descriptive statistics are in *SI Appendix,* Table S1 and an overview of surveys used in *SI Appendix,* Table S2.

### Temperature and SRBs in Sub-Saharan Africa and India.

**Fig. 2.**
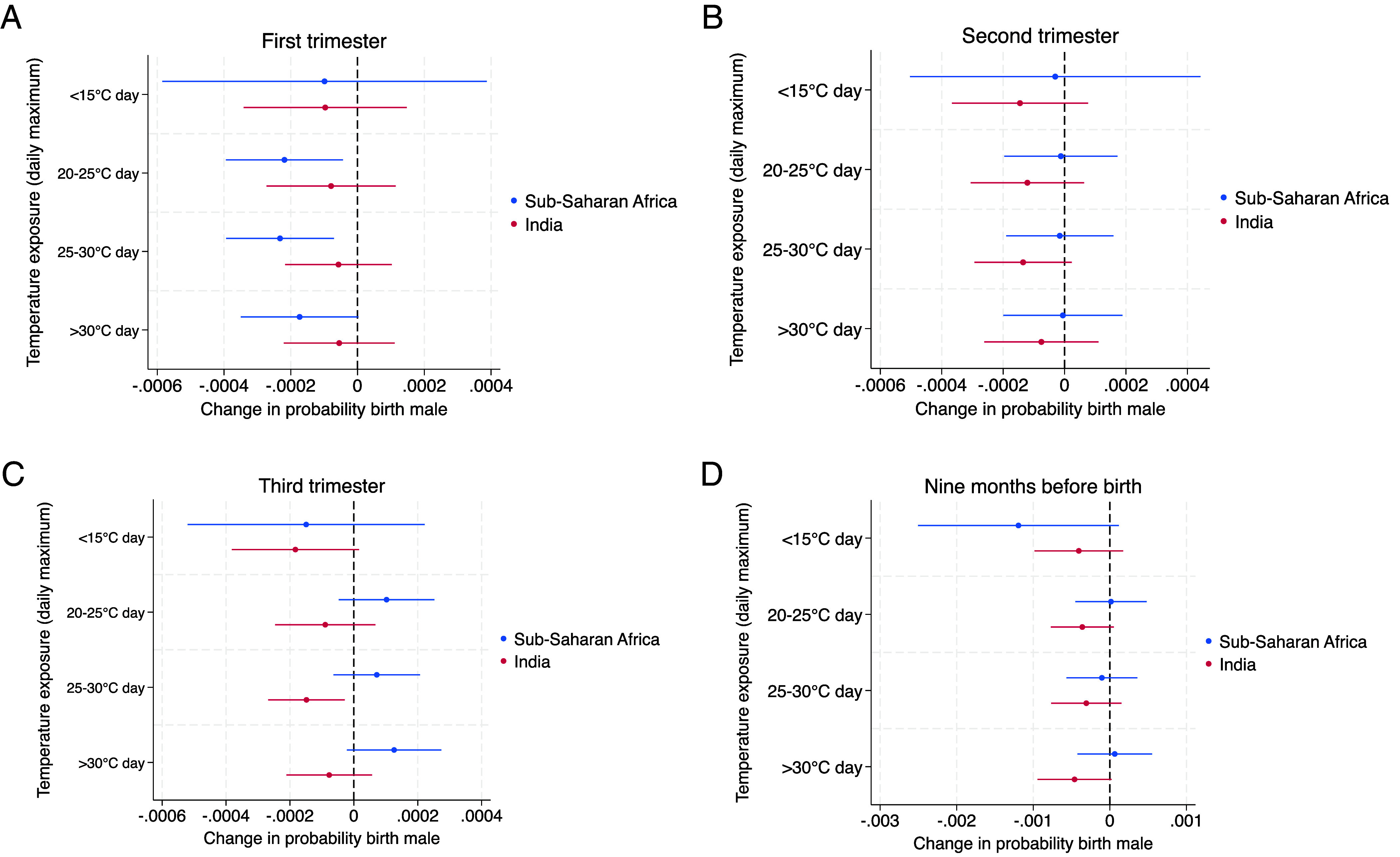
Associations between temperature in the approximate gestational period and male birth, estimated separately for sub-Saharan Africa and India. Coefficients (dots) indicate the change in the probability of the birth being male, with 95% CIs (lines), for one additional day in the approximate gestational trimester where the daily maximum temperature falls into the specified temperature bin. (*A*–*C*) show the association between temperature exposure in each gestational trimester and male birth. (*D*) Shows the association for temperature exposure in the month nine months before the birth month. A detailed description of methods is available in *Materials and Methods*, and detailed results in *SI Appendix,* Tables S3 and S4.

Fig. 2 shows the results from two fixed effects linear probability models of the outcome of male birth on temperatures at different absolute thresholds in each gestational trimester, estimated separately for the sub-Saharan Africa and India sample (*Materials and Methods*). The coefficients indicate the change in the probability of the birth being male for an additional day in the trimester where the daily maximum temperature falls into the specified temperature range (bin) in reference to a 15 to 20 °C d. The models control for region-specific seasonality and annual trends and shocks and exclude births by mothers who were not residents at the PSU location in the year prior to birth. Our results suggest that gestational exposure to days with a maximum temperature above 20 °C is associated with decreases in male birth probability in both sub-Saharan Africa and India, and these effects vary by trimester. Detailed results and effect changes, expressed as the change in the number of male births per 100 female births, are available in *SI Appendix,* Table S3.

In the first trimester (Fig. 2*A*), the estimated coefficients for both sub-Saharan Africa and India all indicate a negative relationship between <15 °C and >20 °C temperature days and male birth probability, though they are only statistically significant for sub-Saharan Africa. Here, an additional day in the first trimester with a daily maximum temperature of 20 to 25 °C decreases the probability of male birth by 0.022 percentage points (or 0.41 percentage points for a 1-SD predictor change, SD = 18.8, *P* = 0.014), a 25 to 30 °C d by 0.023 percentage points (or 0.57 percentage points for a 1-SD predictor change, SD = 24.5, *P* = 0.005), and a >30 °C d by 0.017 percentage points (or 0.6 percentage points for a 1-SD predictor change, SD = 34.8, *P* = 0.054).

Our analysis using monthly lags instead of trimester exposures indicates which approximate gestational months are sensitive to exposure (detailed results in *SI Appendix*, Table S4). The results show that the first-trimester reductions in sub-Saharan Africa appear particularly seven months before birth. The magnitude of effects is substantially larger in the analysis with aggregated trimester exposures, at −0.052 percentage points for an additional 20 to 25 °C d (or −0.34 percentage points for a 1-SD predictor change, SD = 6.7, *P* = 0.032) and −0.051 percentage points for a 25 to 30 °C d (or −0.46 percentage points for a 1-SD predictor change, SD = 9.1, *P* = 0.035).

While in the India sample, the analysis with trimester exposures does not show statistically significant first-trimester effects, there is an indication of male birth reductions for exposure around nine months before birth, with marginally significant effects (Fig. 2*D*). For pregnancies with a standard duration of 40 wk or nine months, the 9-mo lag approximately corresponds to exposure around weeks one to four of gestation, but for shorter pregnancies the exposure might include conception.^§^

In the second trimester, our results in Fig. 2*B* suggest further differences between sub-Saharan Africa and India. We do not identify temperature effects on birth sex in sub-Saharan Africa, where effect estimates are very small and statistically insignificant (−0.001 to 0.003 percentage points for a 1-SD change, *P* = 0.9; 0.897; 0.861; 0.955). However, in India, our results indicate a negative relationship between temperature exposure and birth sex. The effect of a 25 to 30 °C d is marginally statistically significant and indicates a lower male birth probability by 0.014 percentage points (or 0.26 percentage points for a 1-SD change, SD = 19.3, *P* = 0.094). The coefficients for 20 to 25 °C and >30 °C d are of similar magnitude (0.19 and 0.25 percentage points for a 1-SD change) but fail to reach statistical significance (p = 0.198; 0.425).

Again, disaggregating trimester effects by monthly exposures (*SI Appendix*, Table S4), the second-trimester male birth reductions in India appear to be driven by exposures six and five months before birth, with a magnitude of −0.02 to −0.46 percentage points for a 1-SD change in the predictor (*P* > 0.05). By contrast, in the adjacent monthly lags—seven and four months before birth—temperatures for the bins above 25°C indicate an insignificant weak and positive relationship. These findings are suggestive of a temperature response that is concentrated in a short and specific time window of six and five months before birth (or approximately 13 to 20 wk of gestation for a standard pregnancy duration of 40 wk or nine months^¶^), with no response thereafter.

Fig. 2*C* shows that in the third trimester, negative temperature effects on male birth probability appear in India, but not in sub-Saharan Africa. One additional 25 to 30 °C d in the third trimester decreases male births in India by 0.015 percentage points (or 0.35 percentage points for a 1-SD change, SD = 23.4, *P* = 0.015). The other temperature bins fail to reach statistical significance but consistently indicate a negative relationship. Examining results for monthly exposures (*SI Appendix*, Table S4), the third-trimester effects in India are driven particularly by exposure two months before birth. Two months before birth, a 20 to 25 °C d is associated with a lower male birth probability by 0.034 percentage points (or 0.22 percentage points for a 1-SD change, SD = 6.5, *P* = 0.042) and a 25 to 30 °C d with a 0.037 percentage points reduction (or 0.31 percentage points for a 1-SD change, SD = 8.3, *P* = 0.045). In sub-Saharan Africa, our results show statistically insignificant positive temperature effects.

Our findings reveal male birth reductions in response to heat in both sub-Saharan Africa and India, but sensitive time windows of gestational exposure differ between the two regions. Across neither sample do we find evidence of a gradient in the sex response by heat intensity, i.e., the magnitude of SRB reductions is not larger for hotter temperature days. However, once the temperature coefficients are translated into the effect for a 1-SD change in the exposure variable (instead of the effect for one additional day of the exposure variable), temperatures at higher intensity tend to have larger effects on male birth probability. This means that since days of extreme heat intensity of 25 to 30 °C and >30 °C occur more frequently in the two pooled samples, such high intensities play an important role in shaping the sex ratio. *SI Appendix,* Tables S3 and S4 help interpret the magnitude of effects as absolute changes in male per 100 female births. In sub-Saharan Africa, a 1-SD increase in >30 °C d in the first trimester decreases the SRB from 103.54 to 101.08 male births per 100 female births, a reduction by 2.47 male births per 100 female births. In India, a 1-SD increase in 25 to 30 °C d in the second trimester decreases the SRB from 109.95 to 108.81 male births per 100 female births, a reduction by 1.15 male births per 100 female births.

In addition, we find no evidence that geographic differences in the frequency of heat days significantly shape the curve of the temperature–sex relationship. While our models account for time-invariant differences between geographic regions with the use of region-level fixed effects, we test adaptation within each sample specifically. For this purpose, we use alternative exposure variables reflecting PSU-specific relative thresholds that are based on deciles for each PSU’s long-term temperature distribution from 1997 to 2022 (*SI Appendix*, Fig. S2). The results show that in India, the relative thresholds reveal no statistically significant association between temperature and birth sex. In sub-Saharan Africa, some bins in the first trimester (20th to 30th and 50th to 60th, and 80th to 100th percentile days) induce statistically significant male birth reductions. Third-trimester effects are all negative but not consistently significant either. The magnitude of coefficients across bins does not suggest a dose–response by heat intensity. Hence, our findings suggest that the sex ratio at birth may respond to absolute temperature intensity consistently across geographic locations and that location-specific adaptation to extreme heat plays a minor role.

Sub-Saharan Africa’s first-trimester reductions in male births are robust to the inclusion of mother fixed effects that control for all time-invariant health, social, cultural, geographic, financial, and other factors that vary by the mother (*SI Appendix*, Fig. S3). India’s apparent second-trimester reductions in male births, which were not statistically significant in Fig. 2*B*, disappear when including mother fixed effects, suggesting that here characteristics that vary by the mother influence the relationship.

Our back-of-the-envelope calculation suggests that future temperature changes based on different projection scenarios would not decrease the SRB in sub-Saharan Africa and India under global warming (*SI Appendix*, Table S5). This pattern emerges due to the threshold response of the sex ratio, with SRB reductions emerging from 20 °C upward. In sub-Saharan Africa and India, the most significant future shift in temperature will occur in extreme heat days above 30 °C, rather than increases in above 20 °C from below 20 °C.

### Vulnerable Population Subgroups in Sub-Saharan Africa.

**Fig. 3.**
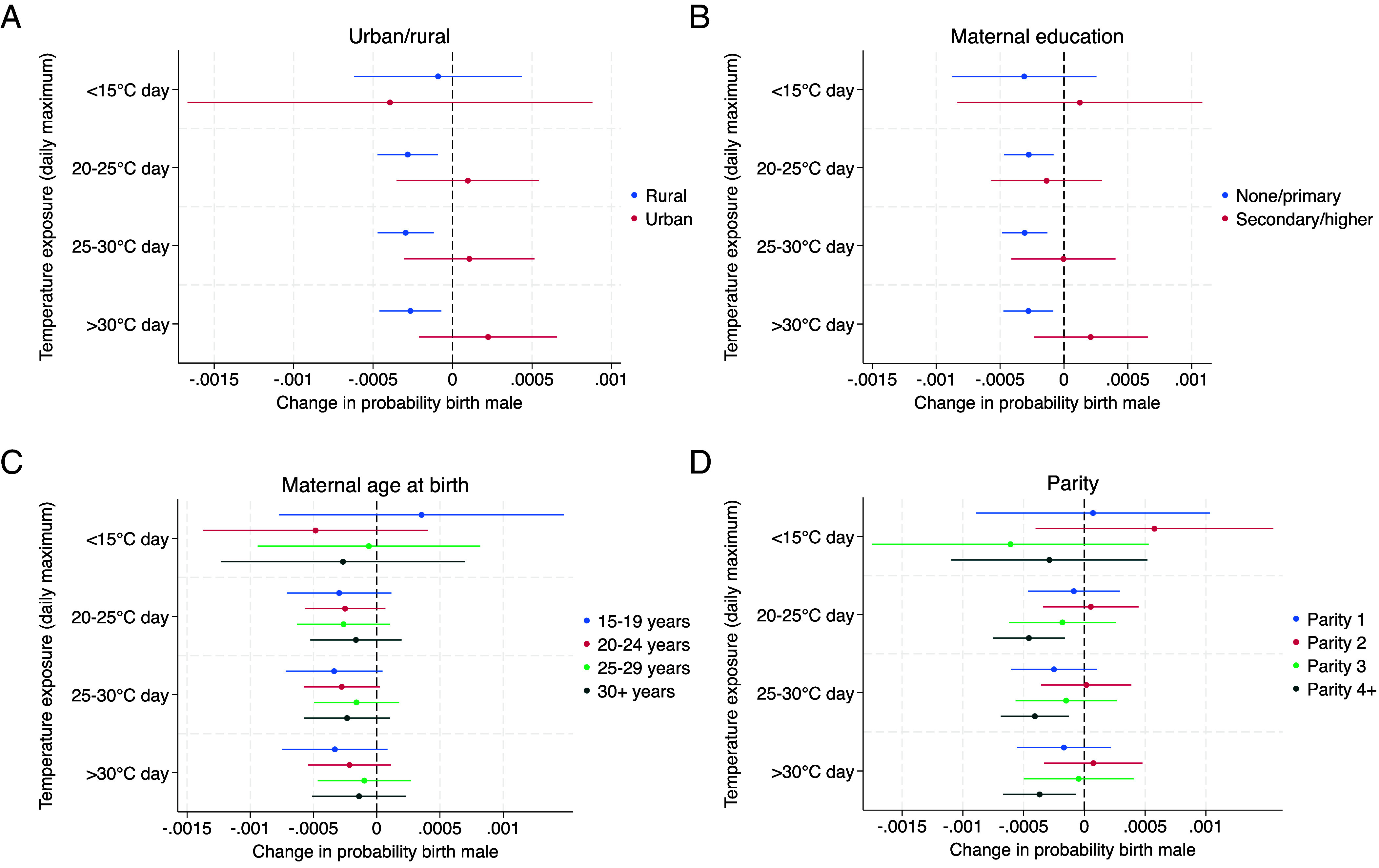
Associations between temperature in the approximate first trimester and male birth in sub-Saharan Africa, estimated separately for each sociodemographic subgroup. Coefficients (dots) indicate the change in the probability of the birth being male, with 95% CIs (lines), for one additional day in the approximate first trimester where the daily maximum temperature falls into the specified temperature bin. The panels show the association for births in urban/rural classifications of the cluster of the mother’s residence (*A*), for births by mothers with no or primary and secondary or higher educational attainment (*B*), for births by mothers at different completed maternal ages at birth (*C*), and by birth order (*D*). A detailed description of methods is available in *Materials and Methods*.

Based on our finding of a negative relationship between heat exposure in the first trimester and male birth in sub-Saharan Africa, we further investigate demographic heterogeneity in the temperature–sex relationship to identify vulnerable population subgroups. Fig. 3 shows the results for first-trimester temperature effects from a separate regression for each population subgroup by the rural/urban classification of the cluster location, maternal educational attainment and age at birth, and the child’s birth order. The results suggest that different sociodemographic factors may shape vulnerability to heat exposure in sub-Saharan Africa (for results for India, see *SI Appendix*, Fig. S4).

Fig. 3*A* indicates that births by mothers residing in rural areas have a lower probability of being male in response to hot temperatures. A 20 to 25 °C d in the first trimester is associated with a 0.028 percentage point decrease in male birth probability (or 0.54 percentage points for a 1-SD change, SD = 19.3, *P* = 0.004), a 25 to 30 °C d with a 0.03 percentage point decrease (or 0.72 percentage points for a 1-SD change, SD = 24.6, *P* = 0.001), and a >30 °C d with a 0.027 percentage point decrease (or 0.93 percentage points for a 1-SD change, SD = 35.1, *P* = 0.008). For urban areas, we observe a nonsignificant and very small positive relationship for temperatures above 20 °C, suggesting that temperature does not affect the SRB in urban areas.

Fig. 3*B* compares temperature effects between births by mothers with no or primary education and by those with secondary or higher education, showing that only the former group exhibits a lower male birth probability. Mothers with no or primary educational attainment show male birth reductions by 0.028 percentage points for a 20 to 25 °C d (or 0.52 percentage points for a 1-SD change, SD = 18.7, *P* = 0.005), by 0.031 percentage points for a 25 to 30 °C d (or 0.75 percentage points for a 1-SD change, SD = 24.3, *P* = 0.001), and by 0.028 percentage points for a >30 °C d (or 0.98 percentage points for a 1-SD change, SD = 35.18, *P* = 0.005). The estimate for a <15 °C d also indicates a negative relationship but is not statistically significant (*P* = 0.281). By contrast, we detect no relationships between temperatures and birth sex for births by mothers with secondary or higher education.

Fig. 3*C* shows that we find no remarkable difference in the temperature–sex relationship by maternal age at birth. While for all age groups the temperature effect estimates indicate a negative relationship with male births, none of the estimates are statistically significant.

The results by birth order in Fig. 3*D* indicate a U-shaped relationship, where parities four and higher show decreases in male births. These decreases are statistically significant only for parities four and higher, though estimates are also negative for first births. Fourth and higher births are less likely to be male by 0.046 percentage points for one additional 20 to 25 °C d (or 0.84 percentage points for a 1-SD change, SD = 18.4, *P* = 0.003), 0.041 percentage points for one 25 to 30 °C d (or 0.99 percentage points for a 1-SD change, SD = 24.4, *P* = 0.004), and 0.037 for one >30 °C d (or 1.28 percentage points for a 1-SD change, SD = 34.8, *P* = 0.016). These results suggest larger temperature effects on the SRB in high parity births.

In a supplementary analysis using the Köppen-Geiger climate zone classification, we do not find evidence that these first-trimester reductions in sub-Saharan Africa are driven by either tropical, arid, or temperate climate zones in particular (*SI Appendix*, Fig. S5).

### Prenatal Sex-Selection Responses in India.

**Fig. 4.**
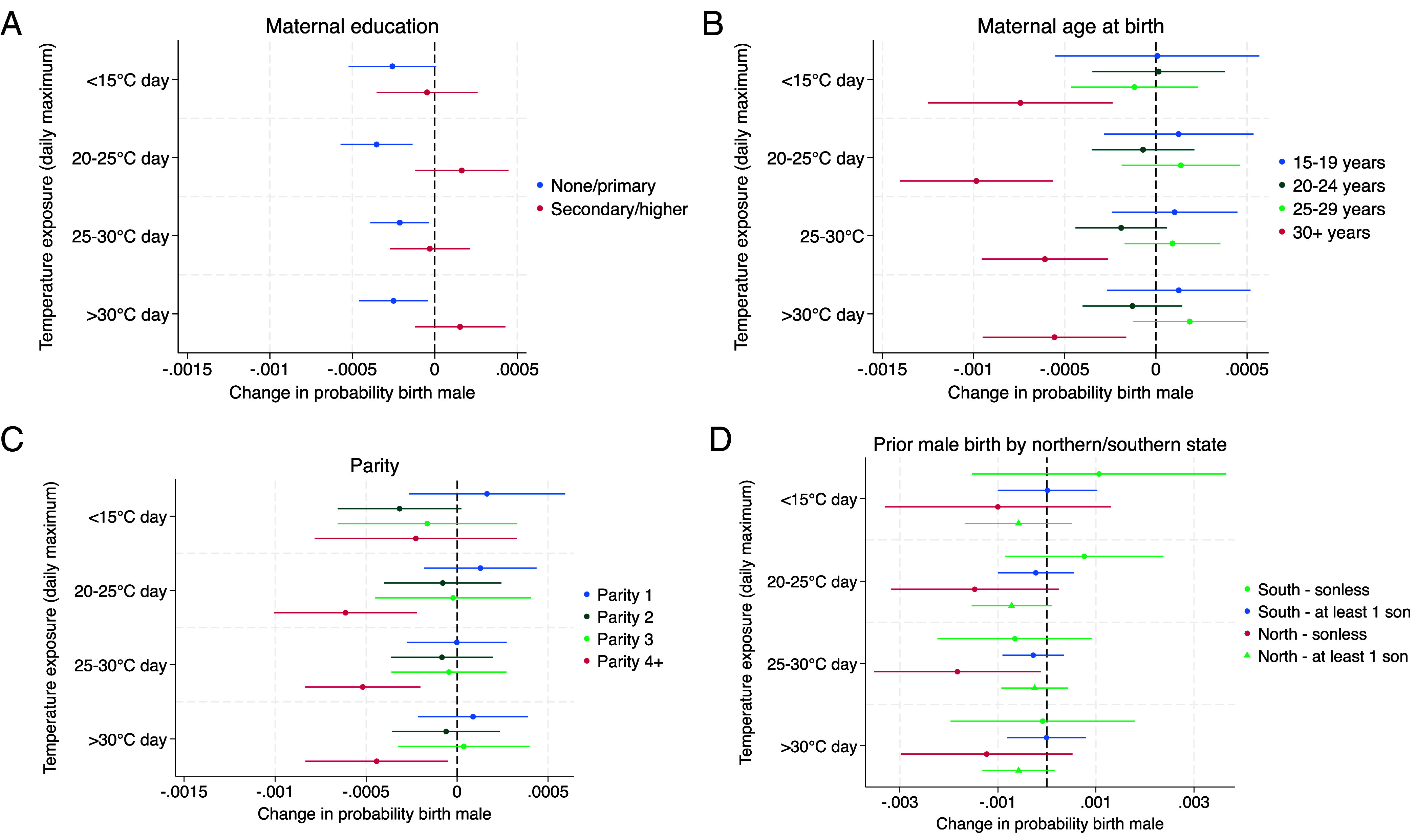
Associations between temperature in the approximate second trimester and male birth in India, estimated separately for each sociodemographic subgroup. Coefficients (dots) indicate the change in the probability of the birth being male, with 95% CIs (lines), for one additional day in the approximate second trimester where the daily maximum temperature falls into the specified temperature bin. The panels show the association for births by mothers with no or primary and secondary or higher educational attainment (*A*), for births by mothers at different completed maternal ages at birth (*B*), by birth order (*C*), and for sonless mothers and mothers with at least 1 previous male birth in northern/southern states of India for birth orders four and higher (*D*). A detailed description of methods is available in *Materials and Methods*.

Next, we examine heterogeneities in the temperature–sex relationship in the second trimester in India analogously for the same sociodemographic characteristics as for the first trimester in sub-Saharan Africa, using a separate regression for each subgroup (Fig. 4).

First, while we find no differences in SRB responses between urban and rural locations (see *SIAppendix*, Fig. S6), we find that births from mothers with little formal education are less male-biased (Fig. 4*A*). Only births from mothers with no or primary formal education have a lower SRB in response to heat, as opposed to births by mothers with at least secondary education, where we observe no changes. The effects for days with a maximum temperature above 20 °C range from −0.021 to −0.035 percentage points for one additional heat day (or −0.4 and −0.81 percentage points for a 1-SD change, *P*-values <0.03).

Furthermore, we find that births from mothers of maternal ages above 30 years and of parities four and higher are significantly less likely to be male and that male birth reductions are strongly concentrated in these groups. As presented in Fig. 4*B*, births with maternal ages above 30, are less likely to be male by 0.056 to 0.099 percentage points for a day above 20 °C (or 1.19 to 1.87 percentage points for a 1-SD change, *P*-values <0.01). Cold temperature below 15 °C also reduces the SRB. For parity, Fig. 4*C* shows that for birth order four and higher, male birth probability is reduced by 0.061 percentage points for a 20 to 25 °C d (1.0 for a 1-SD change, SD = 16.4, *P* = 0.002), 0.052 percentage points for a 25 to 30 °C d (or 0.96 percentage points for a 1-SD change, SD = 18.5, *P* = 0.001), and 0.044 percentage points for a >30 °C d (or 1.45 percentage points for a 1-SD change, SD = 32.9, *P* = 0.027). For lower parities and maternal ages, we do not observe an association between temperatures and SRBs.

Next, we test whether the second-trimester SRB reductions in India are driven by determinants of sex-selective induced abortion practices in India. Culturally, a preference for sons over daughters and its manifestation in male-skewed sex ratios at birth have been shown to be stronger in northern states of India (17, 18, 46). In addition, sex-selective abortion is practiced particularly by women who already have multiple children but no or few male children among them yet (30–32).

Based on these determinants of sex-selective induced abortion prevalence among the Indian population, we additionally test the temperature–sex relationship across four categories (in a separate regression for each): Focusing on births of parities four and higher, we compare births by sonless mothers with births by mothers who previously had at least one male live birth, in both northern and southern states^#^ (Fig. 4*D*). We find a mostly insignificant but strong association between temperatures and SRBs for births by sonless mothers in the North, and no association for the other groups. For births by sonless mothers in the North, we observe a large reduction in male birth probability by 0.183 percentage points for a 25 to 30 °C d (or 2.77 percentage points for a 1-SD change, SD = 15.2, *P* = 0.035). For the other temperature ranges, the effect estimates are similarly large (0.147 and 0.123 percentage points) but fail to reach statistical significance (*P* = 0.091 and 0.167). For comparison, the temperature effects are more than three times larger in this subgroup than the parity differentials we observed in sub-Saharan Africa.

By contrast, SRBs by mothers with at least one previous male birth in the South—potentially the least likely to induce sex-selective abortion—do not respond to temperature (*P* > 0.3). Similarly, we identify no impact of temperature on the SRBs by sonless mothers in the South, and mothers with at least one prior male birth in the North.

Contrasting these results with analogous models for sub-Saharan Africa as the placebo, we identify no second-trimester temperature–sex relationship in any of the subgroups by these maternal and child characteristics there (*SI Appendix*, Fig. S7). Although the coefficients for parity are not statistically significant, their direction is the opposite pattern compared to India: Instead of the large decreases in male births among high parities as seen in India, in sub-Saharan Africa, we observe an increase.

The negative relationship between heat and male birth probability in the second trimester is not stratified by climate zones of India (*SI Appendix*, Fig. S8). Temperature during pregnancy is not associated with the number of antenatal care visits in India, making this an unlikely mechanism through which temperature affects the SRB in India (*SI Appendix*, Fig. S9), though we can only test quantum (i.e., the number of visits) not tempo (e.g., postponement of visits) effects.

## Discussion

We present evidence that temperature before birth is associated with SRBs. Our results show a negative relationship between temperatures above >20 °C and SRBs (i.e., fewer male births relative to female births) in both sub-Saharan Africa and India. However, we find substantive differences between the two regions. In sub-Saharan Africa, heat exposure in the first trimester decreases the SRB. The decreases are driven by births of mothers who reside in rural locations, have no or primary formal education, and are concentrated in birth orders four and higher. In India, by contrast, where sex-selective induced abortions against girls because of son preference have led to a significant male-skew in SRBs (6), we find that heat exposure in the second trimester decreases SRBs. Here, reductions are driven by high parities and high maternal age births. In addition, we find some indication that large SRB decreases occur in births of parities four and higher by sonless mothers in northern states of India.

Our findings indicate that both biological health responses and behavioral responses explain SRB reductions under heat. In sub-Saharan Africa, we explore social vulnerabilities to heat exposure that may be driven by direct physiological impacts on either conception or pregnancy survival (9, 11, 13, 14). As information on gestational length is only available in the more recent DHS, we are not able to disentangle whether male birth reductions are driven by lower male conception probability under heat or intensified in utero mortality, but our analysis of monthly exposures points to the latter. We find no differences between climate zones, potentially pointing to the relevance of a direct physiological response across geographic conditions. In addition, it is also likely that indirect health and behavioral mechanisms, such as temperature impacts on agricultural productivity and income generation, disease, and nutrition play a role (37, 38, 47). In India, the concentrated timing of the shock (approx. 13th gestational week onward), the heterogeneity pattern by sociodemographic characteristics, the magnitude of the effects observed for subgroups of the Indian population, and the cultural differences in son preference between India and sub-Saharan Africa suggest that fewer sex-selective induced abortions against girls take place when temperatures are above 20 °C, resulting in a lower SRB.

The mechanisms that explain SRB reductions in India in the context of potential changes in sex-selective abortion deserve further attention. We find no evidence that higher temperature hinders mothers from attending antenatal care visits. This makes a lack of access to health facilities during heat episodes a less likely mechanism to explain the SRB reductions, though with the data used we are unable to test whether postponement of visits in a crucial time window could explain the effect. Heat-induced income losses could impact whether mothers can afford sex-selective abortions in private clinics. Income losses are unlikely to arise from crop failure at mildly hot temperature levels, which are already associated with SRB changes. But income losses could arise from heat-related labor hour losses in the short-term (48). Heat-related work hour losses have been shown to induce large earnings losses for informal sector workers in India (49) and to be particularly pronounced for lower income households in LMICs (50). This would be in line with the stratification we observe by mothers’ socioeconomic status, but does not explain the lack of a gradient by heat intensity levels.

The significant variations in SRBs in sub-Saharan Africa following exposure to high temperatures, influenced by factors such as the mother’s education, rural or urban residence, high parity, and advanced maternal age, indicate that these population subgroups are particularly vulnerable to heat stress. Our findings lend support to the Trivers-Willard hypothesis, which suggests that under adverse conditions during pregnancy—such as wars, terrorist attacks, and natural disasters—mothers are more likely to give birth to girls rather than boys (3). Although the evidence regarding the impact of maternal socioeconomic characteristics on SRBs remains mixed (51, 52), the skewed SRB observed among certain subgroups implies that mothers with lower levels of education who live in rural areas, for example, may have a reduced capacity to cope with extreme heat. As a result, they may struggle to protect their vulnerable male fetuses in utero.

This study indicates that substantively large variations in SRBs are linked with temperature and reveals the curve of the temperature–sex relationship. The effect sizes of our results are large, given typical SRB variations both between and within populations over time (6). Further, our study uncovers which temperature intensities the sex ratio is sensitive to. The findings demonstrate that the sex ratio is less male for temperature above 20 °C. The lack of a gradient by heat intensity above 20 °C stands in contrast to evidence on the effect of temperature on fertility (9, 11, 13), suggesting that while pregnancy loss risk may increase at higher heat intensity, the sex difference in prenatal mortality responses remains stable. Because of this threshold effect, future temperature changes under climate change are unlikely to induce SRB reductions in the samples under study because in these geographies the largest future upward shifts in temperature will occur at temperature above 30 °C, rather than shifting below 20 °C d to above 20 °C d in the future.

SRBs vary even at low levels of heat intensity, indicating that even moderately high temperatures may constitute heat stress for the maternal body and lead to behavioral responses that result in substantial changes in the SRB. At the same time, days of high heat intensity of 25 to 30 °C and >30 °C occur more frequently in our samples than moderately hot days, meaning that higher temperatures have a relatively larger impact on the SRB. Further, our results suggest that specific temperature intensities in *absolute* terms may shape the sex ratio at birth consistently across geographic locations. In addition, we find that the relationship between heat and birth sex does not vary by climate zones. This is in line with evidence indicating that peak heat stress is surprisingly similar across diverse climates today (53). Our sample has little variation in <15 °C d, preventing us from identifying associations between cold temperatures and SRBs, indicated in previous literature using data on the Global North (7).

This study has several limitations. First, the lack of information on gestational length may lead to exposure misclassification and prevent us from differentiating conception and in utero mortality channels, a line of inquiry hitherto unexplored. Second, there may be other climatic exposures that moderate the effect of temperature. While we include rainfall controls, future studies may explore the role of humidity, crop yields, and other environmental factors in explaining SRB variations. Fourth, while our models allow for nonlinearity of temperature effects across different heat intensities, we did not investigate the role of heatwaves. Future research should explore how prolonged heat exposure duration shapes the SRB.

Overall, this study demonstrates that there is a sex-specific response to temperature before birth driven by both biological and behavioral responses that lead to substantial impacts on reproduction and population composition. We invite scientists to further explore the potential role of moderating environmental influences, factors that buffer and increase vulnerability to exposure, and the specific direct and indirect mechanisms that link temperature with SRBs. In addition, climate change attribution analyses may illuminate the role of temperature changes in explaining past SRB variations and projection studies may explore the role of global warming for SRBs in other geographic settings.

## Materials and Methods

### Birth and Population Data.

We use population data from 104 georeferenced Demographic and Health Surveys (DHS) conducted between 2000 to 2022 in 33 sub-Saharan African countries and India (44). The DHS is an important source of information on health and well-being for low- and middle-income country populations, particularly women and children. It provides nationally representative cross-sectional data on women of reproductive age (ages 15 to 45), along with information on live births and maternal characteristics, such as educational attainment and age, among others.

For the purpose of this study, we use the DHS Births Recode files and include only those surveys and births where the primary sampling unit (PSU), defined as a grouping of households typically corresponding to a city block in an urban area or a village in a rural area, is georeferenced with latitude and longitude coordinates. For each live birth, its sex, month, and year of birth are recorded. Using this information on the mother’s place of residence and the child’s time of birth, we extract high-resolution gridded climate data for each pregnancy for the month of its birth and the previous nine months to cover its gestational period. Though the clusters are displaced spatially by up to 10 km to preserve respondents’ anonymity, we link the climate data to the recorded PSU location as temperature values do not vary substantially on this scale. We restrict our sample to births by women aged 15 to 45 at childbirth who did not migrate in the year prior to the childbirth to address exposure misclassification bias. In total, we use 4,958,918 births (2,981,905 in sub-Saharan Africa and 1,977,013 in India) from 819,571 women (*SI Appendix*, Table S1). The sample covers 381 administrative divisions on the first subnational geographical level (i.e., state, province, or equivalent) in sub-Saharan Africa, and 34 states in India (see *SI Appendix*, Table S2 for an overview of the surveys and samples).

The outcome variable is a binary indicator of whether the child born is male. The temperature exposure is based on the observed temperature at the mother’s location for the approximate duration of the pregnancy for the respective child, making this a microlevel linkage for which the regression allows for inference on the sample level.

### Climate Data.

Temperature data were obtained from the National Oceanic and Atmospheric Administration’s CPC Global Unified Temperature datasets (available at https://psl.noaa.gov/) (45). NOAA temperature data adjustments reflect well a global warming trend (54). These gridded data provide global coverage of surface temperature, updated daily since 1979 and projected onto a 0.5 × 0.5 grid (ca. 55 km^2^). For each day in the gestational period of a child, we extract the daily maximum temperature for the grid cell that intersects with the mother’s cluster location.

We define the gestational period as the month of birth and the nine months prior to the birth month, to also include exposure around conception. The third trimester includes the month of birth and its three lags (1-mo, 2-mo, and 3-mo lag), the second trimester the 4-mo, 5-mo, and 6-mo lag, and the first trimester the 7-mo, 8-mo, and 9-mo lag. To address autocorrelation, we also include the 10-mo and 11-mo lag. The treatment variables then are a vector of count variables that indicate for each gestational trimester of a live birth the number of days in the trimester with daily maximum temperatures that fall into a specified temperature bin (<15 °C, 15 to 20 °C, 20 to 25 °C, 25 to 30 °C, >30 °C). This results in 15 treatment variables (five temperature bins for three gestational trimesters), of which we exclude the 15 to 20 °C bins for each trimester from the regression as the reference category. The regression estimates the effect of one additional day in the given exposure trimester where the daily maximum temperature falls into the given 5 °C temperature bin.

By using temperature bins, we allow the temperature–sex relationship to be nonparametric, following current best-practice methodology (55). For example, the effect of an additional >30 °C heat day could differ from a 20 to 25 °C heat day. In contrast to a cruder pregnancy exposure period, the trimester exposures provide more detailed insight into sensitive exposure timings, which we exploit to infer about mechanisms. We present results by trimester instead of monthly exposures to preserve statistical power, particularly in the heterogeneity analyses by maternal and birth characteristics (in *SI Appendix*, Table S4, we present main results on monthly exposures). The DHS includes information on birth dates, provided as the month and year of birth. Only more recent surveys include day of birth information. Since day of birth information is unavailable for most observations in the analysis samples, we approximate the gestational period when constructing exposure measurements, as described above. In reality, however, gestational length may differ substantially across births, with preterm births making up an estimated 12.3% of live births in sub-Saharan Africa in 2010 (56). The sample of surveys where day of birth information is available is too small to identify climatic SRB determinants with microlevel linkages in our empirical strategy, given the variation necessary within and across geographical units.

In alternative specifications (*SI Appendix*, Fig. S3), we use relative exposure variables that are based on decile thresholds for each primary sampling unit’s long-term temperature distribution from 1979 to 2022. While this approach may capture how extreme daily temperatures are given the temperature distribution usually observed at the specific location, the estimates mostly fail to reach statistical significance and do not provide more insight into the curve of the temperature–sex relationship than the used absolute thresholds (5 °C bins).

In addition, we extract monthly precipitation gridded data from the Climatic Research Unit (CRU) at the University of East Anglia (57) to control for the total amount of rainfall at the PSU location measured in each approximate gestational month.

### Effect Modifiers.

We examine heterogeneity in the temperature–sex relationship with DHS information on maternal and birth characteristics. This helps us test hypotheses on vulnerability to heat exposure and investigate mechanisms that explain sex ratio at birth variations. For each subgroup, we run a separate regression. We examine differences by the cluster location’s urban–rural classification, formal educational attainment of the mother (none or primary; secondary or higher), maternal age at birth (ages 15 to 24; 25 to 29; 30+), and birth order (1; 2; 3; 4+). To investigate effects by sibling composition, we construct a binary variable that indicates for births of parity four and higher, whether the mother previously had a male live birth or not (sonless; at least one son). For India, we also examine difference between northern and southern states [the northern states include Bihar, Delhi, Gujarat, Haryana, Himachal Pradesh, Jammu and Kashmir, Madhya Pradesh, Maharashtra, Punjab, Rajasthan, and Uttar Pradesh, after Kashyap and Behrman (18)].

### Estimation Strategy.

We use a linear probability model with fixed effects to estimate the relationship between gestational heat exposure and birth sex in separate regressions for sub-Saharan Africa and India and each population subgroup (*Effect Modifiers*). We employ a region-by-calendar-month-of-birth and a region-by-year-of-birth fixed effect, where the region refers to the first administrative unit on the subnational regional level (e.g., regions, states, etc.). For a child *i* born in subnational region *r* in month *m* in year *y*, the fixed effects model for the effect of gestational temperature exposure on the child’s sex (1 = male) can be expressed as:

[1] $${\mathrm{Male}}_{\mathrm{icrmy}}=\sum_{j}^{J}\sum_{k=0}^{K=11}\beta_{k}^{j}Temperatures_{c,t-k}^{j}+{\mathrm{rain}}_{\mathrm{cm}}+\alpha_{\mathrm{rm}}+\delta_{\mathrm{ry}}+\varepsilon_{\mathrm{icrmy}}.$$

The outcome variable is a binary indicator of whether the child born is male. The temperature exposure is based on the observed temperature at the mother’s location for the approximate duration of the pregnancy for the respective child. The temperature vector captures the daily maximum temperature distribution at the mother’s cluster location, counting the number of days where the daily maximum temperature falls into the temperature bin *j* (<15 °C, 15 to 20 °C, 25 to 30 °C, >30 °C) in the pregnancy trimester (*t−k*, where *k* = 0 up to 3). The regression yields one estimated coefficient for each temperature bin in each trimester. The effect of temperature exposure is estimated for each trimester individually (rather than cumulatively). Each coefficient indicates the effect of one additional day (i.e., a one-unit change) in the gestational trimester *k* where the temperature falls into the temperature bin *j*, relative to a 15 to 20 °C d (as this bin is omitted from the regression as the reference category), on the probability of the born child being male. In the article, we additionally present the effect for a 1-SD increase in the exposure variables to make results comparable across the samples for which we run the regressions separately.

The advantage of 5 °C bins over summary (e.g., mean of daily temperature) and deviation (z-scores) measurements is that a separate estimate is derived for each temperature bin/intensity. This allows the model to capture nonlinearity instead of assuming a linear relationship between heat exposure and the sex ratio. This is important as the effect of a hot >30 °C d on the sex ratio may be different from a milder 20 to 25 °C d, for example. By providing an individual estimate for the effect of temperature for each bin (heat intensity), the approach makes little assumptions about the biological significance of different thresholds. Therefore, the models can reveal the sensitive temperature ranges at which exposure affects the sex ratio. The underlying temperature distribution observed in our samples (which ranged from below 0 °C to above 35 °C) was divided into the specified bins to increase statistical power for below 15 °C and above 30 °C d. The use of 5 °C bins is consistent with existing approaches and findings (9, 11, 58) and derived estimates can be used for projections. The choice of a different reference bin does not shift the sample that is used for the estimation, and the coefficients remain stable relative to each other. This approach is considered a best-practice methodology to capture nonparametric temperature impacts (55) and was first pioneered in the context of temperature impacts on fertility by Barreca et al. (9).

${\mathrm{rain}}_{\mathrm{rm}}$ is a control for the total amount of rainfall in each approximate gestational month (the month of birth and the preceding nine months). This control is included because temperature and rainfall may be correlated over time, as demonstrated in meteorological studies that suggest temperature records should not be interpreted without considering its covariability with precipitation (59, 60). Furthermore, rainfall could also impact the sex ratio at birth by having effects on maternal health through nutrition, income availability, infectious disease, and other factors brought about by rainfall levels that are also linked to humidity levels. Hence, we control for rainfall as a potential confounder of the temperature–sex relationship.

The region-by-month-of-birth fixed effects $\alpha_{\mathrm{rm}}$ control for region-specific seasonal patterns and other observed and unobserved factors that are constant within the month-region unit, as there can be substantial differences in seasonality, climatic conditions, population vulnerability, and SRBs.

The estimation thus captures the effect of temperature differences on the outcome, given the location and time of the year. These fixed effects are key to our causal interpretation: They limits the temperature variation to fluctuations that occur across years (but within the same region and calendar month), making it plausibly random. The region-by-year-of-birth fixed effects $\delta_{\mathrm{ry}}$ control for annually time-varying factors on the subnational regional level. Alternatively, we explored region-year time trend instead of using single years, which yielded equivalent results. $\varepsilon_{\mathrm{irmy}}$ are the equation’s error terms. This fixed effects approach requires sufficient variation between and within place-time units, which we achieve by pooling surveys across sub-Saharan Africa and India in our main specifications. SE are clustered at the region level.

To test the effect of gestational temperature exposure on antenatal care visits in India (*SI Appendix*, Fig. S9), we employ a poisson regression model with region-by-month-of-birth, region-by-year-of-birth, and DHS cluster fixed effects. The cluster fixed effects control for time-invariant characteristics in each community, such as healthcare infrastructure. The exposure variables count the number of days in each temperature bin across the entire approximate gestational period, rather than individually for each trimester, as antenatal visits might be delayed.

## Supplementary Material

Appendix 01 (PDF)

## Data Availability

The population data used in this study originate from the Demographic and Health Surveys (DHS) and cannot be shared, in line with the program’s Terms of Use: https://dhsprogram.com/data/Terms-of-Use.cfm. For research purposes, data users can request access free of charge on the program’s website here: https://dhsprogram.com/Data/ (44). Though the DHS Program was terminated in January 2025 because of USAID funding cuts, an interim grant by the Gates Foundation currently enables data access for a three-year period since July 2025. The daily surface temperature and rainfall data used in this study are publicly available at the National Oceanic and Atmospheric Association (NOAA, dataset CPC Global Unified Temperature): https://psl.noaa.gov/data/gridded/data.cpc.globaltemp.html (45). To facilitate reproducibility, all data cleaning and analysis files have been deposited in a public repository: https://doi.org/10.17605/OSF.IO/Q5T4S (61).

## Footnotes

^*^The sex ratio at birth (the ratio of male to female offspring) is also referred to as the secondary sex ratio. The ratio of male to female offspring at conception is referred to as the primary sex ratio.

^†^Angola, Burkina Faso, Benin, Burundi, Democratic Republic of Congo, Cote d’Ivoire, Cameroon, Ethiopia, Gabon, Ghana, Guinea, Kenya, Comoros, Liberia, Lesotho, Madagascar, Mali, Malawi, Mozambique, Nigeria, Niger, Namibia, Rwanda, Sierra Leone, Senegal, Swaziland, Chad, Togo, Tanzania, Uganda, South Africa, Zambia, Zimbabwe.

^‡^DHS samples are typically stratified by subnational regions (administrative level 1, e.g., province or states) and rural/urban areas. Within each strata, PSUs (clusters) are selected with probability proportional to size. In rural areas, a PSU refers to a village, and in urban areas to a city block, from which 25 to 30 households are then selected through equal probability sampling. The GPS information (latitude and longitude) provided for the PSU allows us to link high-resolution gridded climate data with the survey data on the cluster level.

^§^This is an approximation as most of the Demographic and Health Surveys we use do not include day of birth and gestational length information, only month of birth information. For further details, see *Materials and Methods*.

^¶^If exposure in the month of birth corresponds to gestational weeks 37 to 40, exposure nine months before birth should correspond to weeks one to four, eight months before birth to five to eight weeks of gestation, etc.

^#^Category of northern states: Bihar, Delhi, Gujarat, Haryana, Himachal Pradesh, Jammu and Kashmir, Madhya Pradesh, Maharashtra, Punjab, Rajasthan, and Uttar Pradesh, after Kashyap and Behrman (18).
