# Supplementary material for: Temperature and sex ratios at birth
Source: Proc Natl Acad Sci U S A. 2026 Feb 19;123(8):e2422625123. doi: 10.1073/pnas.2422625123 (PMC12933076; doi:10.1073/pnas.2422625123)
Supplement: Supplementary file 1 — Appendix 01 (PDF) [file pnas.2422625123.sapp.pdf]

PNAS

Supporting Information for
Temperature and Sex Ratios at Birth.

Jasmin Abdel Ghany, Joshua Wilde, Anna Dimitrova, Ridhi Kashyap, Raya Muttarak

Corresponding authors:

Jasmin Abdel Ghany
Email: jasmin.abdelghany@nuffield.ox.ac.uk

Joshua Wilde
Email: josh.wilde@dph.ox.ac.uk

This PDF file includes:

Figures S1 to S9
Tables S1 to S5

Other supporting materials for this manuscript include the following:

/

Figures

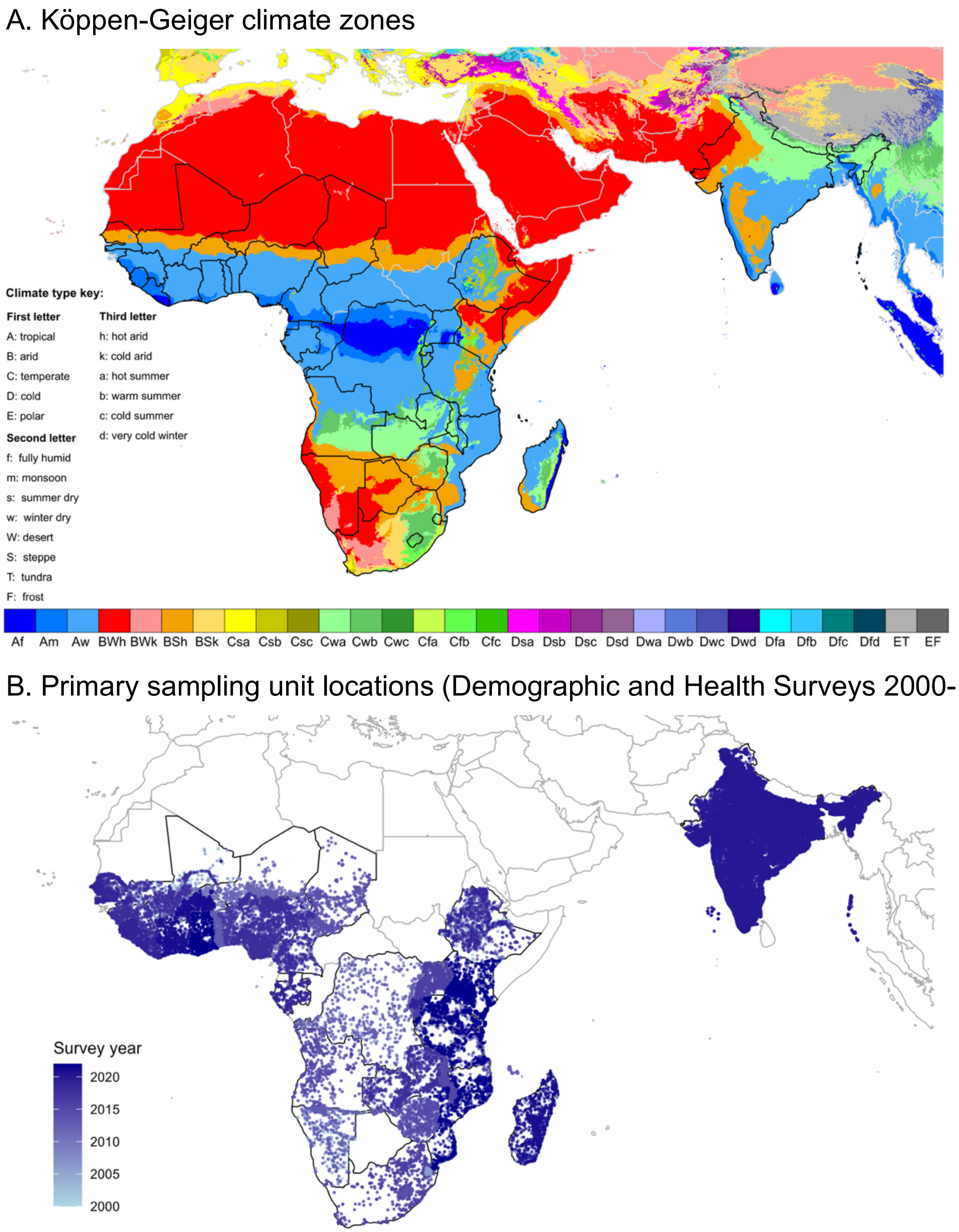

Fig. S1: Coverage of Köppen-Geiger climate zones (Panel A) and location of the primary sampling units included in the sample from the Demographic and Health Surveys in Sub-Saharan Africa and India in 2000-2021 (Panel B).

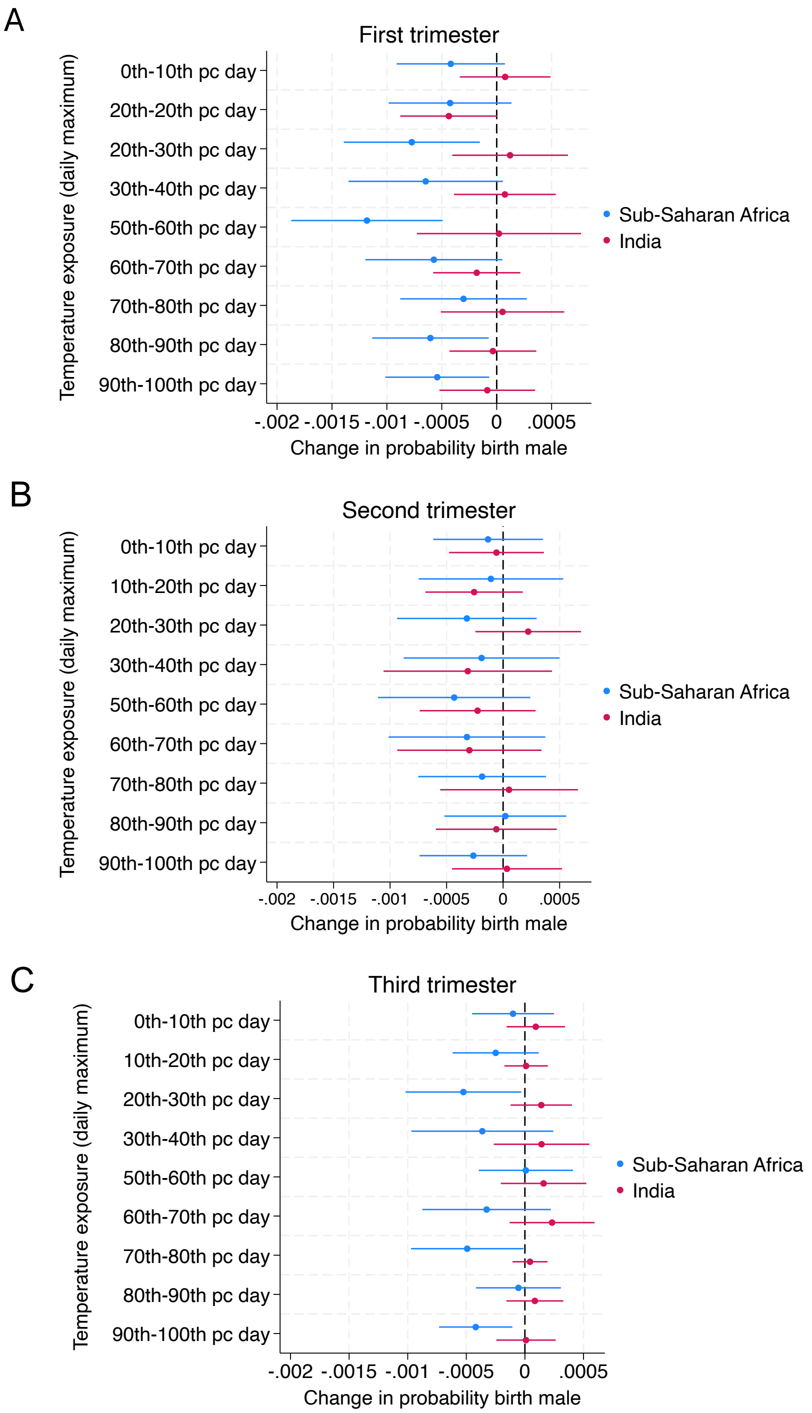

Fig. S2: Associations between temperature in the approximate trimester and male birth from separate regressions for sub-Saharan Africa and India. Coefficients (dots) indicate the change in the probability of the birth being male, with 95% CIs (lines), for one additional day in the approximate trimester where the daily maximum temperature falls into the specified temperature bin. The percentile thresholds are defined based on each primary sampling unit's long-term daily maximum temperature distribution over the years 1979–2022. The panels show the association between temperature exposure for each gestational trimester. A detailed description of methods is available in ***Materials and Methods***.

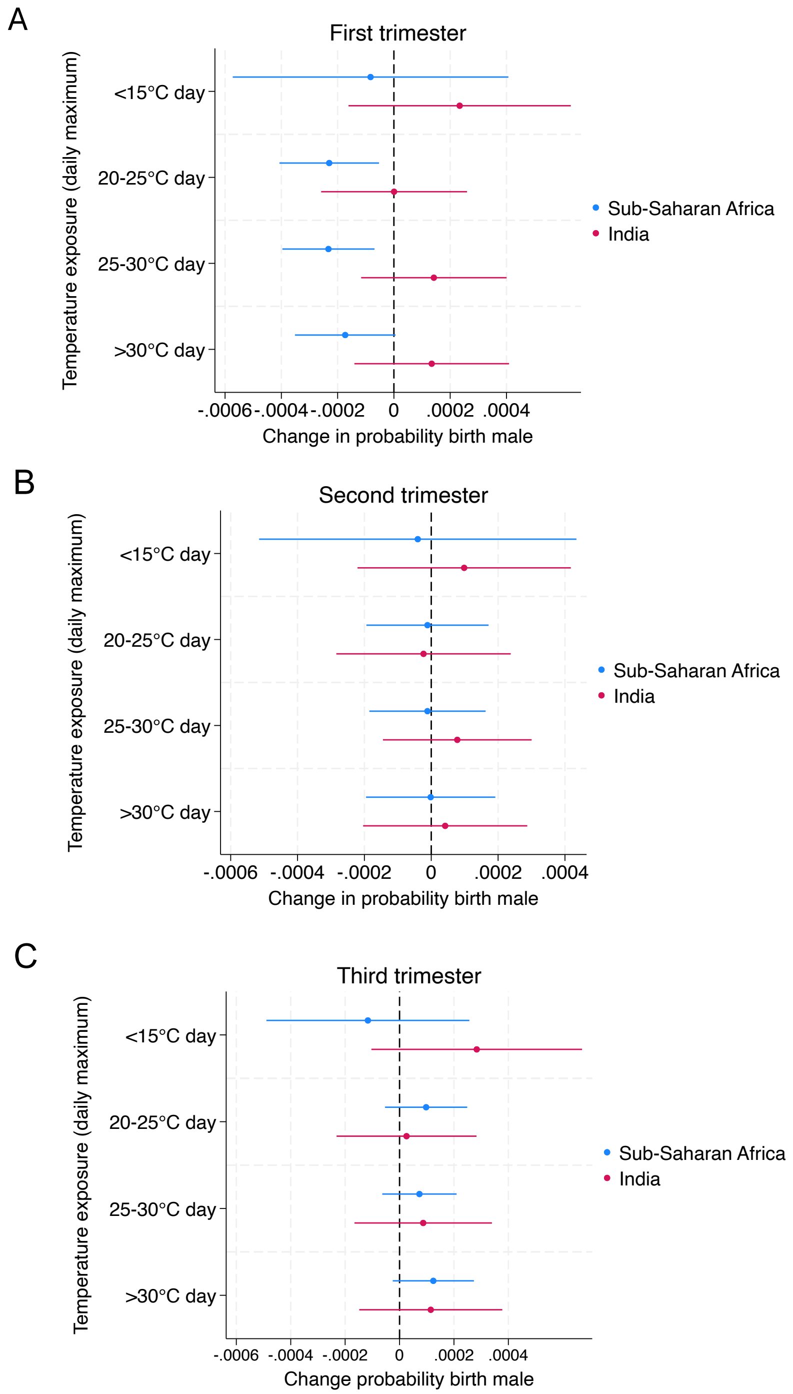

Fig. S3. Associations between temperature in the approximate trimester and male birth, estimated separately for Sub-Saharan Africa and India. Fixed effects include a mother fixed effect in addition to subnational region-by-month-of-birth and region-by-year-of-birth fixed effects. Coefficients (dots) indicate the change in the probability of the birth being male, with 95% CIs (lines), for one additional day in the approximate trimester where the daily maximum temperature falls into the specified temperature bin. The panels A-C show the association between temperature exposure for each gestational trimester. A detailed description of methods is available in ***Materials and Methods***.

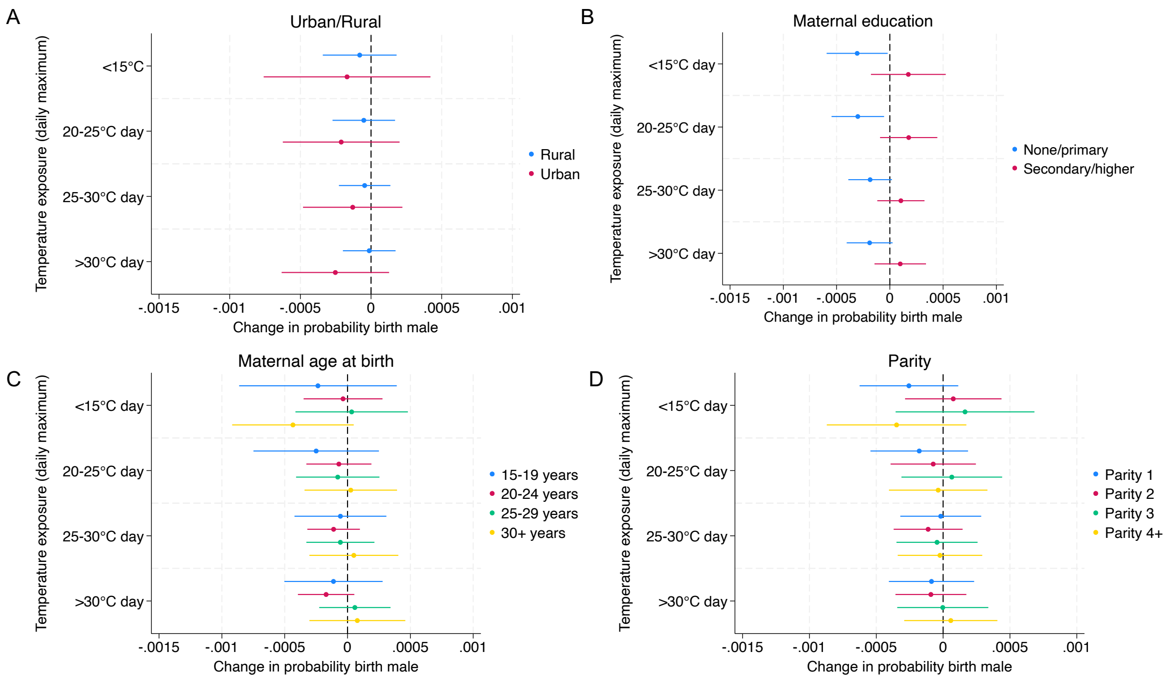

Fig. S4: Associations between temperature in the approximate first trimester and male birth in India by sociodemographic characteristics.

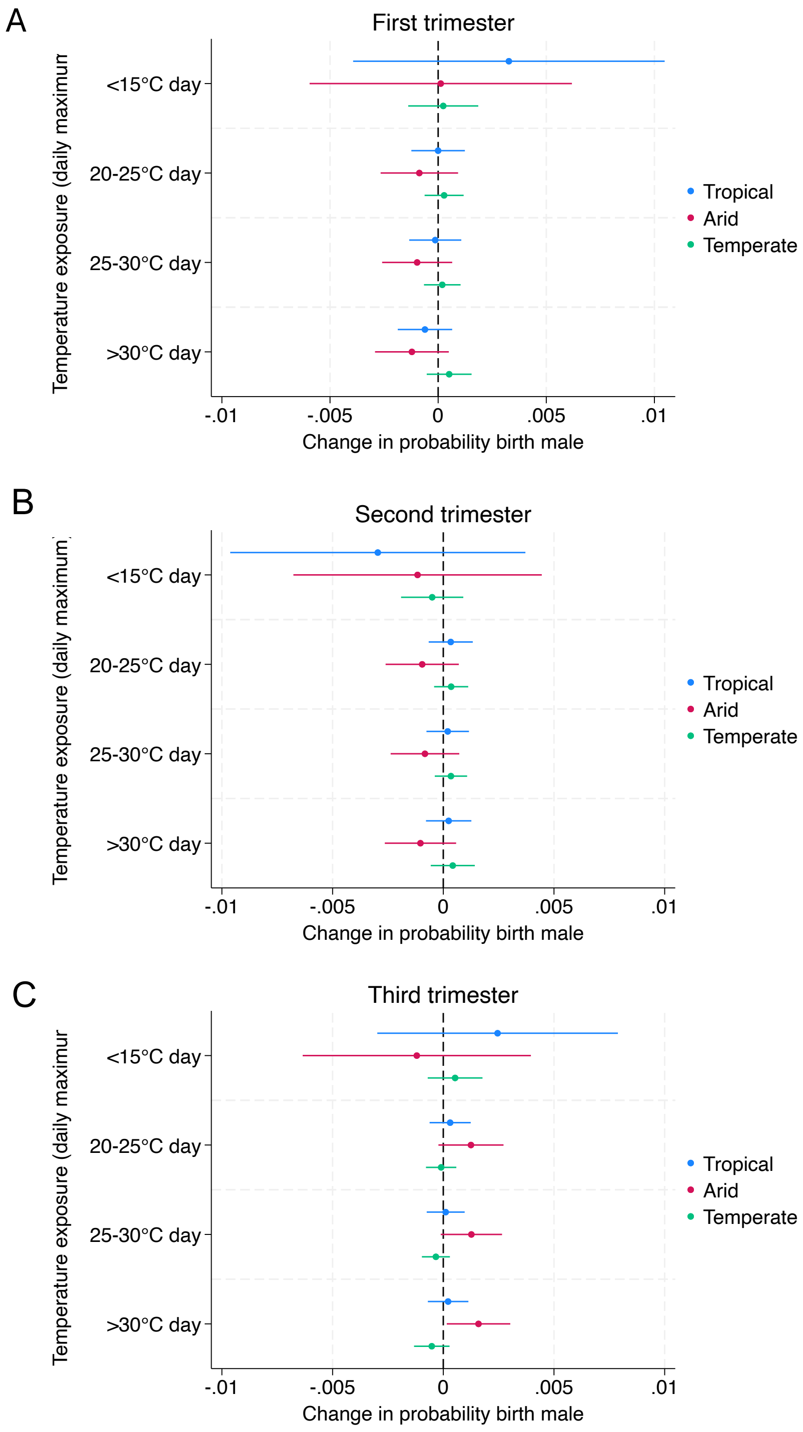

Fig. S5. Associations between temperature in each approximate trimester and male birth in Sub-Saharan Africa, estimated separately for each climate zone (Köppen-Geiger classification). Coefficients (dots) indicate the change in the probability of the birth being male, with 95% CIs (lines), for one additional day in the approximate trimester where the daily maximum temperature falls into the specified temperature bin. The panels show the association between temperature exposure in the first (Panel A), second (Panel B), and third (Panel C) gestational trimester and male birth. The climate zone classification is based on the location of the DHS cluster where the mother resides. Number of observations by climate zone: Tropical N=578,851, Arid N=301,792, Temperate N=212,782. A detailed description of methods is available in ***Materials and Methods***.

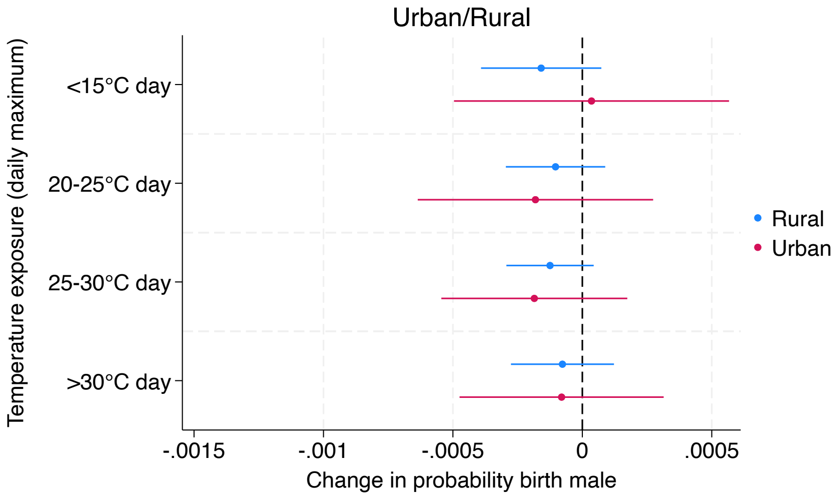

Fig. S6: Associations between temperature in the approximate second trimester and male birth in India by urban/rural classification of the cluster where the mother is resident. Coefficients (dots) indicate the change in the probability of the birth being male, with 95% CIs (lines), for one additional day in the approximate second trimester where the daily maximum temperature falls into the specified temperature bin.

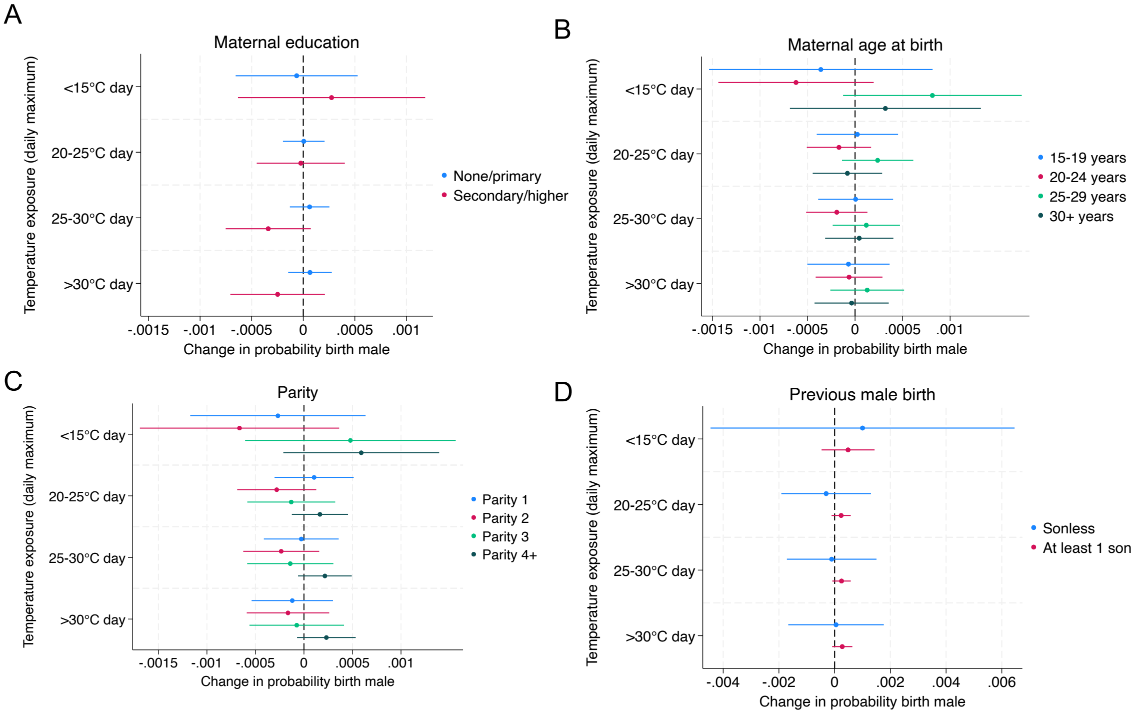

Fig. S7: Associations between temperature in the approximate second trimester and male birth in sub-Saharan Africa by sociodemographic characteristics. Coefficients (dots) indicate the change in the probability of the birth being male, with 95% CIs (lines), for one additional day in the approximate second trimester where the daily maximum temperature falls into the specified temperature bin.

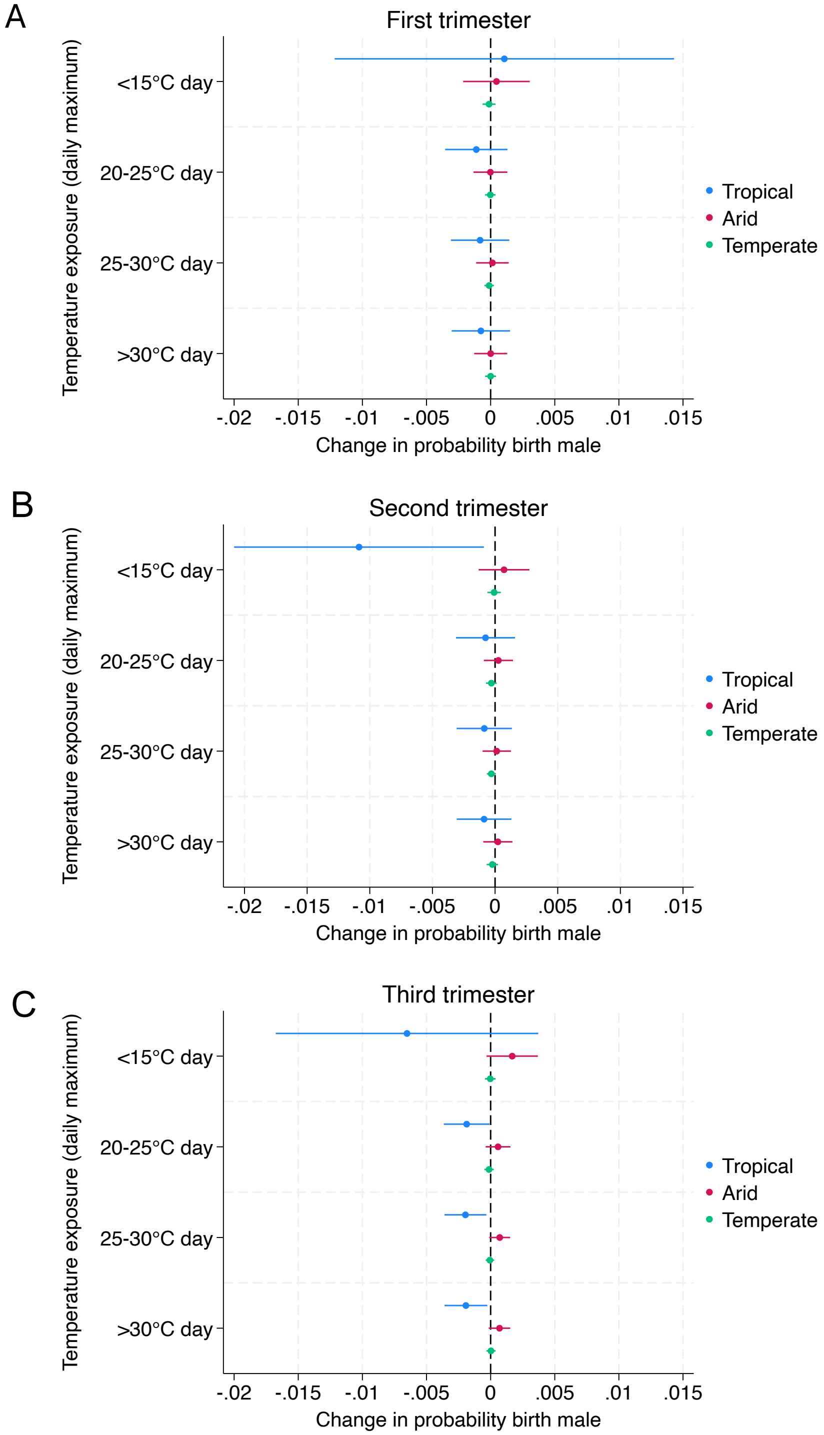

Fig. S8. Associations between temperature in each approximate trimester and male birth in India, estimated separately for each climate zone (Köppen-Geiger classification). Coefficients (dots) indicate the change in the probability of the birth being male, with 95% CIs (lines), for one additional day in the approximate trimester where the daily maximum temperature falls into the specified temperature bin. The panels show the association between temperature exposure in the first (Panel A), second (Panel B), and third (Panel C) gestational trimester and male birth. The climate zone classification is based on the location of the DHS cluster where the mother resides. Number of observations by climate zone: Tropical N=560,486, Arid N=375,469, Temperate N=887,554. A detailed description of methods is available in ***Materials and Methods***.

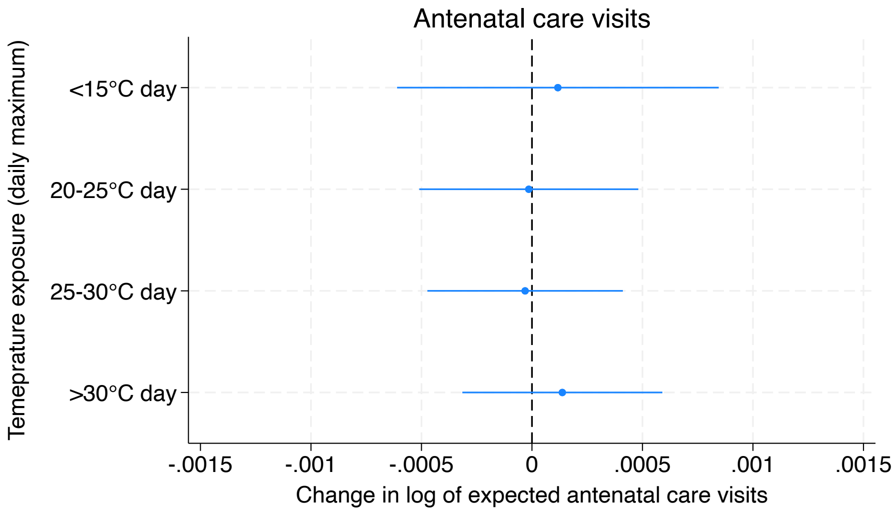

Fig. S9. Associations between temperature in the approximate gestational period and the number of antenatal care visits for the pregnancy in India. Estimates from a poisson regression with region-by-month, region-by-year, and DHS cluster fixed effects. Coefficients (dots) indicate the change in the log of the expected number of antenatal care visits, with 95% CIs (lines), for one additional day in the gestational period where the daily maximum temperature falls into the specified temperature bin. N=294,979, Pseudo R2= 0.304, Residual df = 407. A detailed description of methods is available in ***Materials and Methods***.

Tables

Table S1: Summary statistics (Data source: Demographic and Health Surveys 2000-2021)

Variable	Frequency (Proportion)/ Mean (SD)	
**Births & Mothers**	**Sub-Saharan Africa**	**India**
Total births (N)	2,981,905	1,977,013
Male births	1,516,787 (50.87 %)	1,035,441 (52.37 %)
Female births	1,465,118 (49.13 %)	941,572 (47.63 %)
Mothers	28,890	790,681
**Temperature exposure**		
Daily maximum (month of birth)	29.97°C (4.78)	30.33°C (6.18)
1st trimester below 15°C days	0.41 (3.22)	2.12 (10.44)
1st trimester 15-20°C days	3.3 (8.73)	4.83 (10.63)
1st trimester 20-25°C days	13.1 (18.78)	11.09 (16.13)
1st trimester 25-30°C days	31.13 (24.53)	24.07 (19.30)
1st trimester above 30°C days	44.24 (34.75)	50.27 (32.60)
2nd trimester below 15°C days	0.41 (3.2)	2.39 (11.42)
2nd trimester 15-20°C days	3.3 (8.73)	4.83 (10.63)
2nd trimester 20-25°C days	12.9 (18.68)	10.87 (15.98)
2nd trimester 25-30°C days	30.21 (24.38)	23.65 (19.34)
2nd trimester above 30°C days	45.29 (34.89)	50.66 (32.59)
3rd trimester below 15°C days	0.57 (4.22)	3.12 (14.61)
3rd trimester 15-20°C days	3.30 (8.73)	4.83 (10.63)
3rd trimester 20-25°C days	17.25 (24.45)	14.05 (19.47)
3rd trimester 25-30°C days	39.56 (31.05)	30.22 (23.37)
3rd trimester above 30°C days	60.98 (45.85)	69.52 (41.07)
**Maternal Age**		
15-24 years	1,516,795 (50.87 %)	1,183,522 (59.86 %)
25-29 years	729,890 (24.48 %)	534,008 (27.01 %)
30+ years	735,220 (24.66 %)	259,483 (13.12 %)
**Birth order**		
1st births	673,929 (22.6 %)	595,721 (30.13 %)
2nd births	613,885 (20.59 %)	637,299 (32.24 %)
3rd births	499,991 (16.77 %)	376,025 (19.02 %)
4th and higher births	1,194,100 (40.04%)	367,968 (18.61%)
**Education**		
No education	1,529,044 (51.28 %)	895,784 (45.10 %)
Primary education	983,840 (32.99 %)	315,819 (15.96 %)
Secondary/higher education	468,875 (15.72 %)	765,410 (38.71 %)
Missing	146 (0.00 %)	0 (0.00%)
**Urban/Rural**		
Rural	2,201,859 (73.84 %)	1,580,189 (79.93 %)
Urban	780,046 (26.16 %)	396,824 (20.07 %)
**Northern and southern states (India)**		
Northern states	–	824,272 (41.69 %)
Southern states	–	1,152,741 (58.31 %)
**Survey and geographic information**		
Total number of surveys	102	2
Total number of countries	33	1
Subnational administrative regions (GEO-LEV1)	381	34
DHS clusters	2,269	56,658

Table S2: Overview of Demographic and Health Surveys used in the analysis

Country	DHS Survey ID	Obs. in sample	Percent of pooled sample
Angola	AO2011MIS	21,021	0.70%
	AO2015DHS	39,746	1.33%
Burkina Faso	BF2003DHS	30,919	1.04%
	BF2010DHS	51,856	1.74%
	BF2021DHS	40,793	1.37%
Benin	BJ2001DHS	13,189	0.44%
	BJ2012DHS	42,740	1.43%
	BJ2017DHS	39,359	1.32%
Burundi	BU2010DHS	24,169	0.81%
	BU2016DHS	44,788	1.50%
Democratic Republic of Congo	CD2007DHS	22,148	0.74%
	CD2013DHS	52,754	1.77%
Cote d'Ivoire	CI2012DHS	25,969	0.87%
	CI2021DHS	38,395	1.29%
Cameroon	CM2004DHS	18,037	0.60%
	CM2011DHS	39,638	1.33%
	CM2018DHS	31,701	1.06%
Ethiopia	ET2000DHS	31,891	1.07%
	ET2005DHS	31,522	1.06%
	ET2011DHS	41,819	1.40%
	ET2016DHS	38,657	1.30%
	ET2019DHS	21,403	0.72%
Gabon	GA2012DHS	20,498	0.69%
	GA2019DHS	10,814	0.36%
Ghana	GH2003DHS	9,432	0.32%
	GH2008DHS	7,189	0.24%
	GH2014DHS	21,716	0.73%
	GH2022DHS	22,716	0.76%
Guinea	GN2005DHS	21,468	0.72%
	GN2012DHS	26,397	0.89%
	GN2018DHS	27,702	0.93%
Kenya	KE2008DHS	14,351	0.48%
	KE2014DHS	66,698	2.24%
	KE2022DHS	56,009	1.88%
Comoros	KM2012DHS	7,365	0.25%
Liberia	LB2007DHS	13,728	0.46%
	LB2009MIS	7,952	0.27%
	LB2013DHS	27,690	0.93%
	LB2019DHS	21,094	0.71%
Lesotho	LS2004DHS	10,278	0.34%
	LS2009DHS	8,907	0.30%
	LS2014DHS	11,110	0.37%
Madagascar	MD2008DHS	37,478	1.26%
	MD2021DHS	44,380	1.49%
Mali	ML2001DHS	34,793	1.17%
	ML2006DHS	42,654	1.43%
	ML2012DHS	32,544	1.09%
	ML2018DHS	27,782	0.93%
Malawai	MW2000DHS	23,598	0.79%
	MW2004DHS	23,117	0.78%
	MW2010DHS	51,849	1.74%
	MW2015DHS	66,361	2.23%
Mozambique	MZ2011DHS	35,968	1.21%
	MZ2015AIS	5,862	0.20%
	MZ2022DHS	31,524	1.06%
Nigeria	NG2003DHS	15,940	0.53%
	NG2008DHS	74,347	2.49%
	NG2010MIS	18,500	0.62%
	NG2013DHS	113,466	3.81%
	NG2018DHS	101,472	3.40%
Niger	NI2012DHS	43,004	1.44%
Namibia	NM2000DHS	9,155	0.31%
	NM2006DHS	10,910	0.37%
	NM2013DHS	17,387	0.58%
Rwanda	RW2005DHS	19,642	0.66%
	RW2008DHS	17,890	0.60%

	RW2010DHS	32,201	1.08%
	RW2015DHS	29,716	1.00%
	RW2019DHS	30,464	1.02%
Sierra Leone	SL2008DHS	13,404	0.45%
	SL2013DHS	43,123	1.45%
	SL2019DHS	34,580	1.16%
Senegal	SN2005DHS	28,185	0.95%
	SN2008MIS	41,351	1.39%
	SN2010DHS	39,350	1.32%
	SN2012DHS	21,583	0.72%
	SN2014DHS	20,987	0.70%
	SN2015DHS	22,283	0.75%
	SN2016DHS	21,923	0.74%
	SN2017DHS	41,010	1.38%
	SN2018DHS	22,971	0.77%
	SN2019DHS	20,759	0.70%
Swaziland	SZ2006DHS	5,792	0.19%
Chad	TD2014DHS	66,193	2.22%
Togo	TG2013DHS	25,455	0.85%
Tanzania	TZ2007AIS	14,074	0.47%
	TZ2010DHS	26,063	0.87%
	TZ2012AIS	9,379	0.31%
	TZ2015DHS	33,693	1.13%
	TZ2022DHS	30,344	1.02%
Uganda	UG2000DHS	9,959	0.33%
	UG2006DHS	14,365	0.48%
	UG2009MIS	13,144	0.44%
	UG2011DHS	27,061	0.91%
	UG2016DHS	54,442	1.83%
South Africa	ZA2016DHS	13,280	0.45%
Zambia	ZM2007DHS	11,749	0.39%
	ZM2013DHS	27,566	0.92%
	ZM2018DHS	36,340	1.22%
	ZW2005DHS	14,068	0.47%
Zimbabwe	ZW2010DHS	17,832	0.60%
	ZW2015DHS	11,965	0.40%
India	IA2015DHS	986,996	49.92 %
	IA2020DHS	990,017	50.08%

Table S3: Main regression (trimesters)

Variable	Trim	Beta	SE	p	SD of variable in sample	Beta*SD	Baseline SRB (% male)	SRB (% male) + Beta	Baseline SRB (males: 100 females)	SRB after 1-SD change (males to 100 females)	Difference in male births per 100 females
**Sub-Saharan Africa** (N= 2,981,905, Adjusted R2=0.0002)											
<15°C day	(3)	-0.00015	0.00019	0.430	4.22	-0.00063	50.87%	50.81%	103.54	103.28	-0.260
<15°C day	(2)	-0.00003	0.00024	0.900	3.20	-0.00010	50.87%	50.86%	103.54	103.50	-0.040
<15°C day	(1)	-0.00010	0.00025	0.690	3.22	-0.00032	50.87%	50.84%	103.54	103.41	-0.132
20-25°C day	(3)	0.00010	0.00008	0.180	24.45	0.00250	50.87%	51.12%	103.54	104.58	1.043
20-25°C day	(2)	-0.00001	0.00009	0.897	18.68	-0.00023	50.87%	50.85%	103.54	103.45	-0.094
20-25°C day	(1)	-0.00022	0.00009	0.014	18.78	-0.00411	50.87%	50.46%	103.54	101.85	-1.688
25-30°C day	(3)	0.00007	0.00007	0.299	31.05	0.00223	50.87%	51.09%	103.54	104.47	0.928
25-30°C day	(2)	-0.00002	0.00009	0.861	24.38	-0.00038	50.87%	50.83%	103.54	103.38	-0.157
25-30°C day	(1)	-0.00023	0.00008	0.005	24.53	-0.00569	50.87%	50.30%	103.54	101.21	-2.332
>30°C day	(3)	0.00013	0.00008	0.095	45.85	0.00578	50.87%	51.45%	103.54	105.96	2.422
>30°C day	(2)	-0.00001	0.00010	0.955	34.89	-0.00019	50.87%	50.85%	103.54	103.46	-0.080
>30°C day	(1)	-0.00017	0.00009	0.054	34.75	-0.00603	50.87%	50.27%	103.54	101.08	-2.466
**India** (N= 1,977,013, Adjusted R2= 0.0006)											
<15°C day	(3)	-0.00018	0.00010	0.072	14.61	-0.00267	52.37%	52.1%	109.95	108.78	-1.172
<15°C day	(2)	-0.00015	0.00011	0.199	11.42	-0.00166	52.37%	52.2%	109.95	109.22	-0.729
<15°C day	(1)	-0.00010	0.00012	0.437	10.44	-0.00101	52.37%	52.3%	109.95	109.51	-0.444
20-25°C day	(3)	-0.00009	0.00008	0.263	19.47	-0.00174	52.37%	52.2%	109.95	109.19	-0.765
20-25°C day	(2)	-0.00012	0.00009	0.198	15.98	-0.00194	52.37%	52.2%	109.95	109.10	-0.850
20-25°C day	(1)	-0.00008	0.00010	0.421	16.13	-0.00128	52.37%	52.2%	109.95	109.39	-0.562
25-30°C day	(3)	-0.00015	0.00006	0.015	23.37	-0.00347	52.37%	52.0%	109.95	108.44	-1.517
25-30°C day	(2)	-0.00014	0.00008	0.094	19.34	-0.00261	52.37%	52.1%	109.95	108.81	-1.146
25-30°C day	(1)	-0.00006	0.00008	0.484	19.30	-0.00110	52.37%	52.3%	109.95	109.47	-0.484
>30°C day	(3)	-0.00008	0.00007	0.258	41.07	-0.00317	52.37%	52.1%	109.95	108.57	-1.386
>30°C day	(2)	-0.00008	0.00009	0.425	32.59	-0.00246	52.37%	52.1%	109.95	108.87	-1.081
>30°C day	(1)	-0.00005	0.00008	0.517	32.60	-0.00179	52.37%	52.2%	109.95	109.17	-0.785

Table S4: Main regression (monthly lags)

Variable	Exposure month	Beta	SE	p	SD of variable in sample	Beta*SD	Baseline SRB (% male)	SRB (% male) + Beta	Baseline SRB (males: 100 females)	SRB after 1-SD change (males to 100 females)	Difference in male births per 100 females
**Sub-Saharan Africa** (N=2,981,905, Adjusted R2=.0002)											
<15°C day	lag0	0.00038	0.65000	0.513	1.20	0.00045	50.9%	50.9%	103.54	103.73	0.19
<15°C day	lag1	-0.00025	-0.43000	0.668	1.22	-0.00030	50.9%	50.8%	103.54	103.42	-0.13
<15°C day	lag2	-0.00008	-0.13000	0.895	1.23	-0.00010	50.9%	50.9%	103.54	103.50	-0.04
<15°C day	lag3	-0.00060	-1.01000	0.315	1.22	-0.00074	50.9%	50.8%	103.54	103.24	-0.31
<15°C day	lag4	0.00000	0.00000	0.997	1.19	0.00000	50.9%	50.9%	103.54	103.54	0.00
<15°C day	lag5	0.00004	0.06000	0.955	1.18	0.00004	50.9%	50.9%	103.54	103.56	0.02
<15°C day	lag6	-0.00018	-0.29000	0.775	1.18	-0.00022	50.9%	50.8%	103.54	103.45	-0.09
<15°C day	lag7	0.00025	0.39000	0.700	1.18	0.00029	50.9%	50.9%	103.54	103.66	0.12
<15°C day	lag8	0.00041	0.63000	0.530	1.18	0.00049	50.9%	50.9%	103.54	103.74	0.20
<15°C day	lag9	-0.00119	-1.78000	0.075	1.19	-0.00142	50.9%	50.7%	103.54	102.95	-0.59
<15°C day	lag10	0.00054	0.80000	0.425	1.20	0.00065	50.9%	50.9%	103.54	103.81	0.27
<15°C day	lag11	-0.00026	-0.44000	0.663	1.20	-0.00031	50.9%	50.8%	103.54	103.41	-0.13
20-25°C day	lag0	0.00026	1.15000	0.252	6.70	0.00172	50.9%	51.0%	103.54	104.26	0.71
20-25°C day	lag1	-0.00006	-0.26000	0.794	6.71	-0.00043	50.9%	50.8%	103.54	103.36	-0.18
20-25°C day	lag2	0.00024	0.98000	0.326	6.71	0.00158	50.9%	51.0%	103.54	104.20	0.66
20-25°C day	lag3	0.00015	0.60000	0.549	6.69	0.00097	50.9%	51.0%	103.54	103.95	0.40
20-25°C day	lag4	-0.00023	-0.94000	0.346	6.66	-0.00152	50.9%	50.7%	103.54	102.92	-0.63
20-25°C day	lag5	0.00011	0.48000	0.633	6.66	0.00076	50.9%	50.9%	103.54	103.86	0.32
20-25°C day	lag6	0.00012	0.49000	0.622	6.68	0.00079	50.9%	50.9%	103.54	103.87	0.33
20-25°C day	lag7	**-0.00052**	**-2.14000**	**0.032**	**6.69**	**-0.00345**	**50.9%**	**50.5%**	**103.54**	**102.12**	**-1.42**
20-25°C day	lag8	-0.00013	-0.54000	0.589	6.71	-0.00090	50.9%	50.8%	103.54	103.17	-0.37
20-25°C day	lag9	0.00001	0.06000	0.951	6.73	0.00010	50.9%	50.9%	103.54	103.58	0.04
20-25°C day	lag10	-0.00006	-0.26000	0.796	6.73	-0.00042	50.9%	50.8%	103.54	103.37	-0.17
20-25°C day	lag11	-0.00022	-0.94000	0.345	6.72	-0.00151	50.9%	50.7%	103.54	102.92	-0.62
25-30°C day	lag0	0.00008	0.37000	0.710	9.02	0.00073	50.9%	50.9%	103.54	103.85	0.30
25-30°C day	lag1	-0.00001	-0.05000	0.961	8.98	-0.00010	50.9%	50.9%	103.54	103.50	-0.04
25-30°C day	lag2	0.00028	1.16000	0.245	8.95	0.00249	50.9%	51.1%	103.54	104.58	1.04
25-30°C day	lag3	0.00004	0.16000	0.871	8.94	0.00035	50.9%	50.9%	103.54	103.69	0.15
25-30°C day	lag4	-0.00008	-0.33000	0.739	8.95	-0.00073	50.9%	50.8%	103.54	103.24	-0.30
25-30°C day	lag5	0.00003	0.13000	0.895	9.01	0.00028	50.9%	50.9%	103.54	103.66	0.12
25-30°C day	lag6	0.00006	0.27000	0.790	9.04	0.00058	50.9%	50.9%	103.54	103.78	0.24
25-30°C day	lag7	**-0.00051**	**-2.11000**	**0.035**	**9.06**	**-0.00460**	**50.9%**	**50.4%**	**103.54**	**101.65**	**-1.89**
25-30°C day	lag8	-0.00008	-0.33000	0.744	9.10	-0.00071	50.9%	50.8%	103.54	103.25	-0.29
25-30°C day	lag9	-0.00010	-0.44000	0.660	9.11	-0.00095	50.9%	50.8%	103.54	103.15	-0.39
25-30°C day	lag10	0.00005	0.20000	0.843	9.11	0.00044	50.9%	50.9%	103.54	103.72	0.18
25-30°C day	lag11	-0.00028	-1.20000	0.228	9.08	-0.00256	50.9%	50.6%	103.54	102.49	-1.06
>30°C day	lag0	0.00016	0.66000	0.511	12.24	0.00191	50.9%	51.1%	103.54	104.34	0.79
>30°C day	lag1	-0.00003	-0.13000	0.896	12.23	-0.00041	50.9%	50.8%	103.54	103.37	-0.17
>30°C day	lag2	0.00041	1.60000	0.109	12.24	0.00502	50.9%	51.4%	103.54	105.64	2.10
>30°C day	lag3	0.00005	0.21000	0.837	12.24	0.00065	50.9%	50.9%	103.54	103.81	0.27
>30°C day	lag4	-0.00014	-0.55000	0.580	12.20	-0.00175	50.9%	50.7%	103.54	102.82	-0.72
>30°C day	lag5	0.00010	0.39000	0.696	12.20	0.00118	50.9%	51.0%	103.54	104.03	0.49
>30°C day	lag6	0.00010	0.39000	0.698	12.16	0.00121	50.9%	51.0%	103.54	104.04	0.50
>30°C day	lag7	**-0.00045**	**-1.78000**	**0.076**	**12.14**	**-0.00550**	**50.9%**	**50.3%**	**103.54**	**101.29**	**-2.25**
>30°C day	lag8	-0.00015	-0.58000	0.561	12.17	-0.00181	50.9%	50.7%	103.54	102.79	-0.75
>30°C day	lag9	0.00006	0.26000	0.798	12.20	0.00078	50.9%	50.9%	103.54	103.86	0.32
>30°C day	lag10	0.00008	0.29000	0.769	12.26	0.00094	50.9%	51.0%	103.54	103.93	0.39
>30°C day	lag11	-0.00024	-0.98000	0.329	12.24	-0.00298	50.9%	50.6%	103.54	102.31	-1.23
**India** (N= 1,977,013, Adjusted R2=0.0006)											
<15°C day	lag0	-0.00028	0.00032	0.380	3.86	-0.00109	52.37%	52.3%	109.95	109.47	-0.48
<15°C day	lag1	0.00017	0.00029	0.547	4.02	0.00070	52.37%	52.4%	109.95	110.26	0.31
<15°C day	lag2	-0.00056	0.00032	0.082	4.16	-0.00233	52.37%	52.1%	109.95	108.93	-1.02
<15°C day	lag3	-0.00008	0.00031	0.793	4.21	-0.00035	52.37%	52.3%	109.95	109.80	-0.15
<15°C day	lag4	-0.00015	0.00027	0.575	4.18	-0.00064	52.37%	52.3%	109.95	109.67	-0.28
<15°C day	lag5	-0.00038	0.00031	0.220	4.12	-0.00155	52.37%	52.2%	109.95	109.27	-0.68
<15°C day	lag6	-0.00001	0.00033	0.970	4.04	-0.00005	52.37%	52.4%	109.95	109.93	-0.02
<15°C day	lag7	0.00018	0.00029	0.521	3.94	0.00072	52.37%	52.4%	109.95	110.27	0.32
<15°C day	lag8	-0.00015	0.00030	0.619	3.77	-0.00057	52.37%	52.3%	109.95	109.70	-0.25
<15°C day	lag9	-0.00041	0.00029	0.169	3.66	-0.00148	52.37%	52.2%	109.95	109.30	-0.65
<15°C day	lag10	0.00008	0.00031	0.793	3.62	0.00029	52.37%	52.4%	109.95	110.08	0.13
<15°C day	lag11	-0.00045	0.00040	0.265	3.68	-0.00166	52.37%	52.2%	109.95	109.22	-0.73
20-25°C day	lag0	-0.00004	0.00018	0.818	6.51	-0.00027	52.37%	52.3%	109.95	109.83	-0.12
20-25°C day	lag1	0.00003	0.00020	0.862	6.57	0.00022	52.37%	52.4%	109.95	110.05	0.10
20-25°C day	lag2	**-0.00034**	**0.00017**	**0.042**	**6.54**	**-0.00221**	**52.37%**	**52.1%**	**109.95**	**108.98**	**-0.97**
20-25°C day	lag3	-0.00006	0.00020	0.761	6.53	-0.00039	52.37%	52.3%	109.95	109.78	-0.17
20-25°C day	lag4	-0.00016	0.00018	0.383	6.53	-0.00105	52.37%	52.3%	109.95	109.49	-0.46

20-25°C day	lag5	**-0.00004**	**0.00020**	**0.859**	**6.56**	**-0.00023**	**52.37%**	**52.3%**	**109.95**	**109.85**	**-0.10**
20-25°C day	lag6	**-0.00034**	**0.00018**	**0.061**	**6.67**	**-0.00228**	**52.37%**	**52.1%**	**109.95**	**108.95**	**-1.00**
20-25°C day	lag7	0.00006	0.00017	0.727	6.73	0.00040	52.37%	52.4%	109.95	110.13	0.18
20-25°C day	lag8	0.00005	0.00018	0.788	6.65	0.00032	52.37%	52.4%	109.95	110.09	0.14
20-25°C day	lag9	-0.00036	0.00021	0.086	6.55	-0.00236	52.37%	52.1%	109.95	108.92	-1.03
20-25°C day	lag10	0.00000	0.00017	0.979	6.51	-0.00003	52.37%	52.4%	109.95	109.94	-0.01
20-25°C day	lag11	-0.00038	0.00016	0.017	6.45	-0.00245	52.37%	52.1%	109.95	108.88	-1.08
25-30°C day	lag0	-0.00018	0.00018	0.337	8.22	-0.00145	52.37%	52.2%	109.95	109.31	-0.64
25-30°C day	lag1	-0.00001	0.00019	0.939	8.24	-0.00012	52.37%	52.4%	109.95	109.90	-0.05
25-30°C day	lag2	**-0.00037**	**0.00018**	**0.045**	**8.34**	**-0.00310**	**52.37%**	**52.1%**	**109.95**	**108.60**	**-1.36**
25-30°C day	lag3	-0.00014	0.00020	0.471	8.34	-0.00119	52.37%	52.3%	109.95	109.43	-0.52
25-30°C day	lag4	0.00016	0.00019	0.389	8.35	0.00135	52.37%	52.5%	109.95	110.55	0.60
25-30°C day	lag5	**-0.00028**	**0.00020**	**0.162**	**8.37**	**-0.00232**	**52.37%**	**52.1%**	**109.95**	**108.93**	**-1.02**
25-30°C day	lag6	**-0.00042**	**0.00020**	**0.039**	**8.39**	**-0.00355**	**52.37%**	**52.0%**	**109.95**	**108.40**	**-1.55**
25-30°C day	lag7	0.00012	0.00019	0.511	8.37	0.00102	52.37%	52.5%	109.95	110.40	0.45
25-30°C day	lag8	0.00007	0.00019	0.714	8.38	0.00060	52.37%	52.4%	109.95	110.22	0.26
25-30°C day	lag9	-0.00031	0.00023	0.189	8.35	-0.00257	52.37%	52.1%	109.95	108.82	-1.13
25-30°C day	lag10	-0.00004	0.00019	0.830	8.25	-0.00033	52.37%	52.3%	109.95	109.81	-0.14
25-30°C day	lag11	-0.00021	0.00017	0.214	8.27	-0.00176	52.37%	52.2%	109.95	109.18	-0.77
>30°C day	lag0	-0.00005	0.00021	0.818	12.37	-0.00060	52.37%	52.3%	109.95	109.69	-0.27
>30°C day	lag1	-0.00002	0.00022	0.935	12.45	-0.00023	52.37%	52.3%	109.95	109.85	-0.10
>30°C day	lag2	-0.00023	0.00021	0.280	12.46	-0.00288	52.37%	52.1%	109.95	108.69	-1.26
>30°C day	lag3	-0.00018	0.00022	0.419	12.47	-0.00224	52.37%	52.1%	109.95	108.97	-0.98
>30°C day	lag4	0.00031	0.00022	0.161	12.41	0.00381	52.37%	52.8%	109.95	111.64	1.69
>30°C day	lag5	**-0.00037**	**0.00022**	**0.091**	**12.40**	**-0.00461**	**52.37%**	**51.9%**	**109.95**	**107.94**	**-2.01**
>30°C day	lag6	**-0.00030**	**0.00022**	**0.186**	**12.44**	**-0.00368**	**52.37%**	**52.0%**	**109.95**	**108.34**	**-1.61**
>30°C day	lag7	0.00024	0.00022	0.266	12.43	0.00300	52.37%	52.7%	109.95	111.28	1.33
>30°C day	lag8	0.00012	0.00022	0.571	12.40	0.00152	52.37%	52.5%	109.95	110.63	0.67
>30°C day	lag9	-0.00046	0.00025	0.062	12.32	-0.00568	52.37%	51.8%	109.95	107.48	-2.47
>30°C day	lag10	-0.00014	0.00022	0.528	12.32	-0.00168	52.37%	52.2%	109.95	109.21	-0.74
>30°C day	lag11	-0.00008	0.00019	0.666	12.31	-0.00102	52.37%	52.3%	109.95	109.50	-0.45

Table S5: Back-of-the-envelope calculation for prediction of the SRB under climate change scenarios

In the following, we present a back-of-the-envelope calculation to explore the aggregate effects on population sex ratios in the future based on temperature changes induced by climate change. Counterintuitively, the calculation yielded that temperature would increase the SRB (i.e. more males) in the future, despite our finding that extreme heat is associated with reductions in the SRB.

The method involves computing the number of days in each temperature bin in the projection year 2050, in a reference period (2018–2022), and computing the change in days in each bin, and the corresponding change in the SRB.

We first downloaded a temperature distribution for the period 2018 to 2022 from ISIMIP (https://data.isimip.org, gfdlesm4 Model – High quality data suitable for tropical and mid-latitude performance) for daily maximum temperature values. Using the daily maximum temperature of each day in the 2018-2022 period, we then computed the number of days in each temperature bin (<15°C, 15-20°C, 20-25°C, 25-30°C, >30°C). We chose this five-year period as the reference period because these are the last five years that our birth histories cover, and climate change-induced warming has already taken place. Hence, the projection using 2018–2022 as the reference period can be interpreted as a conservative lower bound, given that we our main analysis includes surveys starting from the year 2000. Secondly, we followed the same process to compute the number of days in each bin for the year 2050, based on four different projection scenarios (SSP126, SSP245, SSP370, SSP585).

This means we arrived at four temperature distributions that each indicate for each temperature bin the number of days where the daily maximum temperature fell into that bin. The four temperature distributions refer to the following time periods:

- in 2018 to 2022
- in 2050 for four scenarios currently in use:
    - scenario 1 (SSP1, 2.6°C warming)
    - scenario 2 (SSP2, 4.5°C warming)
    - scenario 3 (SSP3, 7°C warming)
    - scenario 4 (SSP5, 8.5°C warming)

Next, we calculated the change in heat days in each bin from 2018–2022 to 2050. More specifically, for each bin, we compute the difference in the number of days between 2050 and 2018–2022. Hence, the values indicate how many fewer/more days in that bin there will be in 2050, compared to 2018–2022. We do this for each of the four scenarios for 2050. For example, if in scenario 1 for 2050 there are five projected days where the daily maximum temperature will be above 30°C, and in 2018–2022 there were 4 days where the daily maximum temperature was above 30°C, then the difference is one. We conduct this calculation separately for Sub-Saharan Africa and India.

Then, we calculated the expected change in the SRB for each bin, based on the projected number of days in each bin. We do this by multiplying the coefficient for each bin by the change in the number of days for the bin. Since our regression estimates were based on trimesters, we summed the coefficients across trimester for each bin. This assumes that there is a steady flow of pregnancies, where one third of pregnancies is in the first trimester, one third in the second, and one third in the third trimester. We omitted the reference bin, which is also omitted from the regression, hence there is no regression coefficient for this bin. The result indicates the effect of temperature on the dependent variable expressed as percentage point changes for each bin.

Lastly, we calculated the expected SRB in 2050 based on the SRB changes for each bin. To do so, we add the change in the SRB for each bin to the mean of the SRB in our sample. This expected SRB (expressed as the percent of male births) can then be translated into the number of male births per 100 female births. The below tables present these calculations for sub-Saharan Africa (A) and India (B).

A. Temperature shifts in Sub-Saharan Africa

Sub-Saharan Africa – Baseline probability of male birth: .5084 (translates to 103.4174 males per 100 females)

Sub-Saharan Africa: Coefficients

		Trimester	Coefficient	Coefficient for each bin (sum of coefficients across trimesters)
<15°C	day	3	-0.00009	-0.00024
<15°C	day	2	-0.00010	
<15°C	day	1	-0.00005	
20-25°C	day	3	0.00010	-0.00006
20-25°C	day	2	0.00002	
20-25°C	day	1	-0.00018	
25-30°C	day	3	0.00006	-0.00013
25-30°C	day	2	0.00000	
25-30°C	day	1	-0.00019	
>30°C	day	3	0.00012	-0.00003
>30°C	day	2	0.00002	
>30°C	day	1	-0.00017	

Sub-Saharan Africa: Bin distribution in reference period (2018-2022) and projection year (2050) for four different scenarios

Year	Scenario	<15°C days	15-20°C days	20-25°C days	25-30°C days	>30°C days
2018-2022	ssp126	4.205778	6.3842	23.94223	91.14783	239.52
2018-2022	ssp245	4.303931	6.980751	24.59731	92.12021	237.1978
2018-2022	ssp370	4.449353	6.617499	24.0914	91.74233	238.2994
2018-2022	ssp585	4.383591	6.721126	25.11963	94.7036	234.2721
2050	ssp126	4.130992	6.922141	22.44256	81.46817	250.0361
2050	ssp245	3.784555	5.200101	19.14088	81.25171	255.6227
2050	ssp370	3.97371	5.549582	19.78151	77.14938	258.5458
2050	ssp585	3.868248	6.298884	20.03944	75.99531	258.7981

Sub-Saharan Africa: Difference in days per bin between 2050 and 2018-2022

Scenario	<15°C days	15-20°C days	20-25°C days	25-30°C days	>30°C days
ssp126	-0.07479	0.537941	-1.49967	-9.67966	10.51618
ssp245	-0.51938	-1.78065	-5.45643	-10.8685	18.42496
ssp370	-0.47564	-1.06792	-4.30989	-14.5929	20.24636
ssp585	-0.51534	-0.42224	-5.08019	-18.7083	24.52607

Sub-Saharan Africa: SRB change

	<15°C days	20-25°C days	25-30°C days	>30°C days	Δ in P(Male)	Expected P(Male)	Expected SRB	Δ in SRB
ssp126	0.000018	0.000090	0.001258	-0.00032	0.001051	0.509451	103.8532	0.435738
ssp245	0.000125	0.000327	0.001413	-0.00055	0.001312	0.509712	103.9618	0.544421
ssp370	0.000114	0.000259	0.001897	-0.00061	0.001662	0.510062	104.1076	0.690230
ssp585	0.000124	0.000305	0.002432	-0.00074	0.002125	0.510525	104.3004	0.883026

B. Temperature shifts in India

India – Baseline probability of male birth: .525 (translates to 110.5263 males per 100 females)

Coefficients India

		Trimester	Coefficient	Coefficient for each bin (sum of coefficients across trimesters)
<15°C	day	3	-0.00016	-0.00041
<15°C	day	2	-0.00015	
<15°C	day	1	-0.0001	
20-25°C	day	3	-0.00009	-0.00033
20-25°C	day	2	-0.00015	
20-25°C	day	1	-0.00009	
25-30°C	day	3	-0.00013	-0.00036
25-30°C	day	2	-0.00017	

25-30°C	day	1	-0.00006	
>30°C	day	3	-0.00006	-0.00022
>30°C	day	2	-0.00012	
>30°C	day	1	-0.00004	

India: Bin distribution in reference period (2018-2022) and projection year (2050) for four different scenarios

Period	Scenario	<15°C days	15-20°C days	20-25°C days	25-30°C days	>30°C days
2018-2022	ssp126	4.205778	6.3842	23.94223	91.14783	239.52
2018-2022	ssp245	4.303931	6.980751	24.59731	92.12021	237.1978
2018-2022	ssp370	4.449353	6.617499	24.0914	91.74233	238.2994
2018-2022	ssp585	4.383591	6.721126	25.11963	94.7036	234.2721
2050	ssp126	4.130992	6.922141	22.44256	81.46817	250.0361
2050	ssp245	3.784555	5.200101	19.14088	81.25171	255.6227
2050	ssp370	3.97371	5.549582	19.78151	77.14938	258.5458
2050	ssp585	3.868248	6.298884	20.03944	75.99531	258.7981

India: Difference in days per bin between 2050 and 2018-2022

Scenario	<15°C days	15-20°C days	20-25°C days	25-30°C days	>30°C days
ssp126	-0.07479	0.537941	-1.49967	-9.67966	10.51618
ssp245	-0.51938	-1.78065	-5.45643	-10.8685	18.42496
ssp370	-0.47564	-1.06792	-4.30989	-14.5929	20.24636
ssp585	-0.51534	-0.42224	-5.08019	-18.7083	24.52607

India: SRB change

	<15°C days	20-25°C days	25-30°C days	>30°C days	Δ in P(Male)	Expected P(Male)	Expected SRB	Δ in SRB
ssp126	0.000031	0.000495	0.003485	-0.00231	0.001697	0.526697	111.281	0.754683
ssp245	0.000213	0.001801	0.003913	-0.00405	0.001873	0.526873	111.3596	0.833307
ssp370	0.000195	0.001422	0.005253	-0.00445	0.002417	0.527417	111.6028	1.07652
ssp585	0.000211	0.001676	0.006735	-0.0054	0.003227	0.528227	111.9664	1.440034

The result of this back-of-the-envelope calculation yields that the SRB would not decrease but rather increase by 2050 (i.e. more males). This seems counterintuitive given that we find that temperature above 20°C reduces the male birth probability (**Figure 2** in the manuscript). The result is explained by the magnitude of coefficients, which are used to extrapolate SRB changes. In our regression results, the coefficient for the 25-30°C bin is *larger* than the coefficient for the >30°C bin. However, the most significant change in the temperature distribution between 2018–2022 and 2050 is the increase in >30°C days. For this reason, the calculation overall yields an increase in the SRB, even though there will be more >30°C days in the future.

We explored two alternative approaches, which yielded the same result. The result of an SRB increase remains when collapsing the bins above 20°C, as the main shift in the temperature distribution occurs at higher heat intensity levels, rather than at the cut-off of 20°C. The result of an SRB increase is also not driven by collapsing the trimester coefficients.

However, the reason why the >30°C coefficient is estimated as smaller than the 25-30°C is likely imprecision of estimates. While we use the point estimate to calculate the expected SRB change, the coefficients for the bins are not statistically different from each other. From a theoretical perspective, the most likely interpretation of our coefficients is that there is a threshold effect, where temperature above 20°C leads to smaller SRBs. It is unlikely that temperature above 30°C leads to a smaller SRB than temperature between 20°C and 30°C. For this reason, the result of the back-of-the-envelope calculation could be misleading to the reader, as it suggests based on estimate imprecision that SRBs will increase in the future, even though extreme heat (which is increasing in the future) is associated with fewer male births.

At the same time, the lack of an SRB decrease in the future underlines the meaning of the threshold effect from 20°C that we find. Crucially, SRB decreases should be apparent in the future in populations where 1) temperature leads to male-biased pregnancy loss, and 2) where the temperature distribution shifts from below 20°C to more than 20°C. Since in both Sub-Saharan

Africa and India, most days are already above 20°C, future temperature shifts may induce little changes in the SRB.

The back-of-the-envelope calculation, while meant provide an *approximate* indication of how SRBs may develop in the context of climate change, has several important limitations, warranting further projection analysis. First, the expected SRB changes can only be calculated for the pooled Sub-Saharan African and Indian samples. We only provide estimates for the pooled Sub-Saharan African and Indian samples as our main analysis requires a large sample size. We do so because SRBs vary only little over time and between population, which increases sample size requirements. The fixed effects strategy isolates within subnational-region-month variation in temperature, which further increases sample size requirements. While we can only conduct a projection for Sub-Saharan Africa and India, there may be substantial within-sample heterogeneities that may be explored in future studies.

Second, the calculation assumes that the trimester-exposures translate into annual exposures. As described above, we sum the coefficients for each bin across trimesters.

Third, our back-of-the-envelope calculation does not incorporate changes in the SRB that arise from changes in the sex balance, population structure, and dynamics between 2001 and 2049.
